# PartSense-IP: Part-Aware Vision–Language Sensor Fusion for Visual–Semantic Consistency Evaluation of IP Prototypes

**DOI:** 10.3390/s26165052

**Published:** 2026-08-09

**Authors:** Yangfan Feng, Wen Zhao

**Affiliations:** 1 State Key Laboratory for Fine Exploration and Intelligent Development of Coal Resources, China University of Mining & Technology, Beijing 100083, China; atobekigeo@163.com; 2 School of Civil Aviation, Northwestern Polytechnical University, Xi’an 710072, China

**Keywords:** IP prototypes, multi-view RGB-D sensing, vision–language sensor fusion, part-aware perception, visual–semantic consistency, inconsistency localization, 3D prototype inspection

## Abstract

Evaluating whether an intellectual property (IP) prototype faithfully preserves the visual identity and semantic intent of its original concept design is an important yet challenging task in product design and creative prototyping. Existing evaluation practices mainly rely on manual inspection or global image-level similarity comparison, which are subjective, difficult to reproduce, and insufficient for localizing identity-critical deviations. To address this problem, this paper proposes PartSense-IP, a part-aware vision–language sensor fusion framework for visual–semantic consistency evaluation of IP prototypes. The proposed framework takes a 2D concept image, an optional textual design description, and multi-view RGB-D sensor observations of a prototype as inputs. It first constructs a multi-view prototype representation and decomposes both the concept and prototype observations into design-relevant parts. Dense visual features, color and shape descriptors, and vision–language semantic embeddings are then extracted to evaluate part-level consistency. A Part-Aware Visual–Semantic Consistency Fusion (PVCF) algorithm is further developed to integrate shape, color, local visual similarity, semantic alignment, and cross-view stability into a unified IP consistency score. In addition to scalar scoring, PartSense-IP generates localized difference maps, 3D inconsistency visualization, and interpretable design feedback for prototype refinement. Experiments on the proposed IP-ProtoSense evaluation protocol demonstrate that PartSense-IP outperforms representative vision–language, dense-visual, segmentation-based, and 3D multimodal baselines in consistency scoring, inconsistency detection, localization, ablation, and robustness evaluation.

## 1. Introduction

Intellectual property (IP) product design has become increasingly important in character merchandising, cultural creative products, toy design, game-style assets, and digital-physical product prototyping. In a typical design workflow, designers first create a two-dimensional concept artwork that defines the intended visual identity, including the character style, dominant colors, local symbolic elements, decorative patterns, and semantic design intent. The concept is then transformed into a physical or digital prototype through modeling, rendering, manufacturing, or 3D printing. During this transformation process, the prototype may deviate from the original concept in terms of shape, color, texture, local parts, symbolic elements, or semantic identity. For example, a prototype may preserve the overall object category but lose a logo, distort a character’s ears, shift facial components, or weaken the intended toy-like style. These deviations are often evaluated manually by designers or reviewers, which is subjective, time-consuming, and difficult to reproduce.

Recent progress in visual inspection and anomaly detection has provided useful tools for detecting local defects in industrial products. Reconstruction-based and feature-memory-based methods have achieved strong performance in surface anomaly detection and localization [1,2,3,4]. RGB-D and 3D anomaly detection datasets have further extended inspection tasks from 2D appearance defects to geometric defects captured by depth sensors [5]. Beyond local surface defects, logical anomaly detection addresses violations of higher-level constraints concerning component presence, quantity, arrangement, and semantic combination. The MVTec LOCO AD benchmark and its global-context anomaly detection method established a systematic setting for jointly evaluating structural and logical anomalies [6]. Subsequent component-aware methods explicitly model the properties and relationships of object parts. For example, ComAD employs unsupervised component segmentation and component-level metrological features to identify abnormal compositions and relationships [7], while PSAD uses part segmentation and compositional memory representations to detect violations in component quantity, arrangement, and composition [8]. More recently, SALAD introduces semantics-aware composition modeling to preserve both spatial and semantic relationships among object components [9,10].

Vision–language models have also been introduced into industrial anomaly detection to improve zero-shot or few-shot generalization  [11,12,13,14]. However, most existing inspection methods are designed for manufacturing defects such as scratches, dents, missing components, incorrect component counts, or logical arrangement anomalies. Existing inspection approaches include both distribution-based anomaly detection and paired-reference comparison. The former evaluates a test product against learned normal-product statistics, whereas the latter compares it with a registered golden template. Neither setting directly addresses whether an IP prototype preserves the identity-critical appearance and semantic intent of a stylized concept design under multi-view RGB-D sensing. Reference- or golden-template-based comparison represents another directly relevant line of research. Classical industrial inspection systems align a test image with a defect-free reference and detect deviations using image subtraction, normalized cross-correlation, or feature-based template matching [15,16]. Full-reference image-quality metrics, such as the structural similarity index (SSIM), measure structural fidelity between a reference and a test image [17], while learned perceptual metrics such as LPIPS compare their deep visual representations [18].

These methods provide important foundations for paired-reference comparison, but they generally assume that the reference and test images depict spatially aligned observations of the same visual content. In the present task, the reference is a stylized 2D concept artwork, whereas the prototype is represented by multi-view RGB-D observations. Differences in viewpoint, projection, material appearance, illumination, and self-occlusion are therefore not necessarily design inconsistencies. Direct template or perceptual comparison may consequently conflate normal cross-domain variations with identity-critical deviations. Moreover, such metrics do not explicitly model part correspondence, textual design semantics, missing-part logic, or cross-view stability. PartSense-IP extends paired-reference comparison to a part-aware visual–semantic setting under multi-view RGB-D sensing.

In parallel, vision–language models and foundation models have significantly advanced visual–semantic representation learning. CLIP demonstrates that image and text representations can be aligned through large-scale contrastive pretraining [19]. BLIP and BLIP-2 further improve vision–language understanding and generation through bootstrapped pretraining and frozen large model alignment [20,21]. GLIP and Grounding DINO extend language-aware visual understanding to object-level grounding and open-set detection [22,23]. Dense visual representation learning has also progressed rapidly. DINO and DINOv2 show that self-supervised visual transformers can provide strong transferable features for dense image understanding [24,25]. These models are highly relevant to IP prototype evaluation because the task requires both visual comparison and semantic interpretation. Nevertheless, global vision–language similarity alone is insufficient for fine-grained IP consistency evaluation. A prototype may remain globally similar to the reference concept while still losing identity-critical local elements. Therefore, a part-aware and spatially localized evaluation mechanism is required.

Part-level perception is another important direction related to this work. Universal image segmentation models such as Mask2Former and OneFormer provide flexible segmentation architectures for semantic, instance, and panoptic segmentation [26,27]. The Segment Anything Model (SAM) introduces promptable segmentation and demonstrates strong zero-shot transferability to diverse visual domains [28], while HQ-SAM improves fine-grained mask quality for more detailed object structures [29]. SEEM further explores interactive and promptable segmentation with multimodal prompts [30]. These works make it possible to decompose concept images and prototype observations into design-relevant regions. However, segmentation alone does not determine whether each part preserves the intended IP identity. For IP prototypes, local regions such as logos, eyes, ears, accessories, and decorative patterns are not equally important. They should be evaluated according to their visual appearance, semantic role, and cross-view consistency. Existing segmentation models do not explicitly provide such design-oriented consistency scoring or feedback generation.

Another related line of research is 3D perception and 3D multimodal learning. Point-cloud completion and representation learning methods, such as PoinTr, Point-BERT, Point-MAE, and PointNeXt, have improved 3D shape modeling and point-cloud understanding [31,32,33,34]. Inspired by 2D vision–language pretraining, several studies have attempted to transfer language–image knowledge to 3D understanding [35]. PointCLIP and PointCLIP V2 project point clouds into multi-view depth maps and align them with CLIP representations [36,37]. ULIP and ULIP-2 learn unified representations of language, images, and point clouds for 3D understanding [38,39,40]. OpenShape scales multimodal 3D shape representation toward open-world understanding [41]. More recent 3D vision–language models, including 3D-VisTA, 3D-LLM, and Uni3DL, further explore language-grounded 3D understanding and reasoning [42,43,44,45]. These works provide important foundations for aligning 3D data with visual and textual semantics. However, they mainly focus on object recognition, retrieval, grounding, captioning, or general 3D understanding. They do not specifically evaluate whether a sensor-captured prototype preserves the local visual–semantic identity of a 2D IP concept.

Large-scale 3D object datasets also provide useful resources for constructing multi-view sensing benchmarks. Objaverse provides a large collection of annotated 3D objects with diverse categories and visual styles [46]. Amazon Berkeley Objects (ABO) contains product images, metadata, and 3D models for real-world object understanding [47]. Google Scanned Objects provides high-quality 3D scans of household objects for simulation and synthetic perception [48]. OmniObject3D offers real-scanned 3D objects with textured meshes, point clouds, multi-view rendered images, and captured videos [49]. Although these datasets are valuable for 3D perception and simulation, they are not designed for IP prototype consistency evaluation. In particular, they usually do not contain paired 2D concept references, textual design descriptions, controlled prototype deviations, part-level inconsistency labels, human consistency ratings, and designer-oriented feedback annotations. Therefore, a task-specific dataset and evaluation protocol are needed for sensor-based IP prototype assessment.

Based on the above review, three research gaps can be identified. First, existing industrial anomaly detection methods mainly detect surface or structural defects, but they do not model the visual–semantic relationship between a 2D concept artwork and a physical or digital IP prototype. Second, existing vision–language and 3D multimodal methods provide strong global representations, but they do not explicitly evaluate identity-critical local parts under multi-view RGB-D sensing. Third, existing inspection systems usually output anomaly scores or segmentation maps, whereas IP product design requires interpretable feedback that tells designers where the inconsistency occurs and which design attribute should be refined. These gaps motivate a new framework that jointly considers sensor-level prototype observation, part-aware visual representation, vision–language semantic alignment, multi-view consistency fusion, and designer-oriented feedback generation.

To address these challenges, this paper proposes PartSense-IP, a part-aware vision–language sensor fusion framework for visual–semantic consistency evaluation of IP prototypes. The proposed framework takes a concept design, including a 2D concept image and an optional textual design description, together with multi-view RGB-D sensor observations of a prototype as inputs. It decomposes the concept and prototype views into identity-critical parts, extracts dense visual and semantic features, evaluates component-level consistency, and fuses multi-view evidence through the proposed Part-Aware Visual–Semantic Consistency Fusion (PVCF) algorithm. The framework outputs an overall IP consistency score, localized difference maps, 3D inconsistency visualization, and interpretable design feedback.

The main contributions of this paper are summarized as follows:

- We formulate IP product prototype evaluation as a sensor-captured visual–semantic consistency assessment task. Different from conventional image-level similarity comparison or industrial defect detection, the proposed task evaluates whether a physical or digital prototype preserves the visual identity and semantic design intent of a 2D concept artwork under multi-view RGB-D sensing.
- We propose the PartSense-IP framework and the PVCF algorithm as a structured part- and view-level decision framework rather than a new feature-extraction backbone. SAM, DINOv2, and CLIP are used only to provide candidate masks and generic visual–semantic representations. The methodological novelty lies in confidence- and visibility-aware concept-to-prototype part correspondence, null assignment for potentially missing parts, identity-aware part aggregation, and cross-view stability modeling. These operations convert heterogeneous pretrained representations into a unified consistency score, localized 2D and 3D inconsistency maps, and attribute-specific diagnostic feedback.
- We construct an IP-ProtoSense evaluation protocol with concept references, textual descriptions, multi-view RGB-D observations, controlled design deviations, ground-truth inconsistency masks, and human consistency ratings. Extensive experiments, including consistency scoring, inconsistency detection, localization, ablation, robustness evaluation, and 3D-to-2D inconsistency visualization, demonstrate that PartSense-IP provides more accurate, robust, and interpretable prototype assessment than representative vision–language, dense-visual, segmentation-based, and 3D multimodal baselines.

## 2. Proposed PartSense-IP Framework

This section presents the proposed PartSense-IP framework for sensor-captured visual–semantic consistency evaluation of IP prototypes. The main objective is to determine whether a physical or digital product prototype preserves the visual identity and semantic design intent of its original 2D concept artwork. Different from conventional image-level similarity comparison, the proposed framework integrates multi-view RGB-D sensing, part-aware visual perception, and vision–language semantic alignment. The detailed consistency-fusion algorithm and feedback-generation process are further described in Section 3.

PartSense-IP is designed as a backbone-agnostic evaluation framework. The segmentation, dense visual, and vision–language encoders can be implemented using existing pretrained models, such as SAM, DINOv2, and CLIP [24,25,28,29]. These pretrained models themselves are not claimed as technical contributions of this work. Instead, the methodological contribution lies in converting their heterogeneous outputs into matched, design-oriented consistency variables and aggregating these variables hierarchically across components, identity-critical parts, and sensing views. The resulting formulation jointly supports prototype-level scoring, localized inconsistency projection, and attribute-specific diagnosis. Therefore, the underlying encoders can be replaced by future alternatives without altering the proposed task formulation or fusion procedure.

### 2.1. System Model and Overall Workflow

In IP product design, designers usually start from a 2D concept artwork that defines the visual identity of a character, mascot, toy, cultural product, or game-style asset. After several design and fabrication steps, a physical or digital prototype is obtained. However, the prototype may deviate from the original concept in terms of shape, color, texture, local decorative elements, or semantic identity. These deviations are often evaluated manually, which is subjective, time-consuming, and difficult to reproduce. To address this problem, we formulate IP prototype evaluation as a sensor-captured visual–semantic consistency assessment task.

Let the original concept design be represented as

(1) $$C=\left\{I_{c},T_{c}\right\},$$

where $I_{c}$ denotes the 2D concept image, and $T_{c}$ denotes the optional textual design description. The textual description can include key design attributes, such as character style, dominant color, symbolic elements, product category, and semantic identity. For example, $T_{c}$ may describe an IP character as “a cute blue mascot with round ears and a star-shaped logo.”

The product prototype is captured by a multi-view RGB-D sensing system. The sensing observations are denoted as

(2) $$S={\left\{I_{v},D_{v},P_{v}\right\}}_{v=1}^{N},$$

where $I_{v}$ is the RGB image captured from the *v*-th view, $D_{v}$ is the corresponding depth map, $P_{v}$ denotes the camera pose or view metadata, and *N* is the number of sensing views. The views are selected to cover identity-critical and geometry-critical directions, including the front view, side views, back view, top view, and local close-up views when necessary.

Given C and S, the goal of PartSense-IP is to estimate three outputs:

(3) $$\left(S_{\mathrm{IP}},M_{\mathrm{diff}},F_{\mathrm{fb}}\right)=\Phi \left(C,S\right),$$

where $S_{\mathrm{IP}}\in \left[0,1\right]$ is the overall IP consistency score, $M_{\mathrm{diff}}$ is a localized difference map indicating inconsistent regions, and $F_{\mathrm{fb}}$ is an interpretable feedback report for design refinement. A higher $S_{\mathrm{IP}}$ indicates that the prototype better preserves the visual–semantic identity of the original concept artwork.

**Figure 1:**
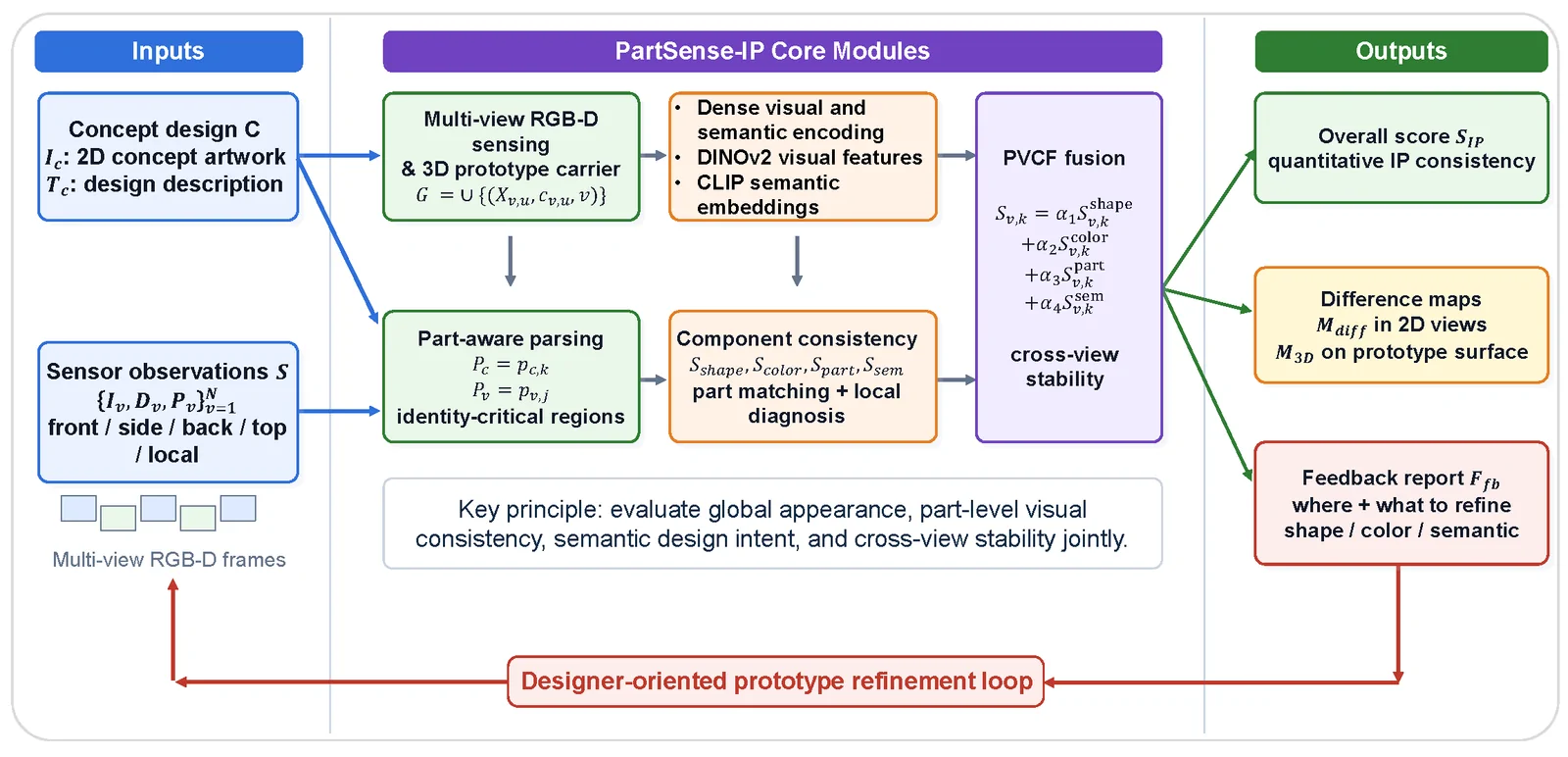
Overall workflow of the proposed PartSense-IP framework. The framework takes the concept design $C=I_{c},T_{c}$ and multi-view RGB-D sensor observations $S={\left\{I_{v},D_{v},P_{v}\right\}}_{v=1}^{N}$ as inputs. The prototype is first represented through multi-view RGB-D sensing and a 3D prototype carrier. Then, concept and prototype observations are decomposed into identity-critical parts, followed by dense visual feature extraction and vision–language semantic alignment. The proposed PVCF module integrates shape, color, part-level visual similarity, semantic consistency, and cross-view stability to generate the overall IP consistency score $S_{IP}$, localized difference maps $M_{\mathrm{diff}}$, 3D inconsistency map $M_{3D}$, and interpretable feedback report $F_{\mathrm{fb}}$. The feedback report supports designer-oriented prototype refinement.

The proposed PartSense-IP framework consists of four major modules, as illustrated in Figure 1. The first module performs multi-view RGB-D sensing and prototype representation. The second module extracts part-aware visual features from both the concept artwork and sensor-captured prototype views. The third module conducts visual–language semantic alignment to measure whether the prototype preserves the design intent. The fourth module fuses the part-level and view-level consistency measurements to generate the final score, difference map, and design feedback.

Specifically, the framework follows the pipeline below. First, the product prototype is captured from multiple viewpoints using an RGB-D sensing setup. The RGB images provide color, texture, and visual appearance information, while the depth maps provide geometric cues for view alignment and 3D fusion. Based on the camera pose information, the sensor observations are projected into a unified prototype representation, such as a point cloud, mesh, or view-aligned multi-view representation. Second, the concept image and the prototype views are parsed into semantic or functional parts. For IP products, these parts may include the head, body, eyes, ears, logo, accessories, surface decorations, and other identity-critical regions. Instead of evaluating the whole image as a single unit, PartSense-IP evaluates whether each design-relevant part is preserved. Third, the framework extracts visual and semantic features from each part. Dense visual features are used to measure local appearance and texture similarity, while vision–language features are used to align the captured prototype with the textual design description. This makes it possible to detect both low-level visual deviations and high-level semantic inconsistency. Finally, a part-aware visual–semantic consistency fusion algorithm is introduced to integrate shape, color, part-level appearance, semantic identity, and multi-view stability into a unified score. The algorithm also localizes inconsistent regions and converts them into human-readable design feedback.

### 2.2. Multi-View RGB-D Sensing and Prototype Representation

The sensing module aims to capture the product prototype from complementary viewpoints. For each view *v*, the RGB-D sensor provides an observation pair $\left(I_{v},D_{v}\right)$. The camera pose $P_{v}$ is used to associate observations across different views. When the camera pose is unavailable, it can be estimated using standard feature matching or visual odometry methods.

The depth map $D_{v}$ is first back-projected into a local 3D point set:

(4) $$x_{v,u}=D_{v}\left(u\right)K^{-1}\tilde{u},$$

where *u* denotes a pixel location, $\tilde{u}$ is its homogeneous coordinate, and *K* is the camera intrinsic matrix. The local 3D point $x_{v,u}$ is transformed into the global coordinate system by

(5) $$X_{v,u}=P_{v}x_{v,u}.$$

By aggregating all valid points from *N* views, a multi-view prototype representation is obtained:

(6) $$G=\overset{N}{\underset{v=1}{⋃}}\left\{\left(X_{v,u},c_{v,u},vs.\right)|u\in \Omega_{v}\right\},$$

where $\Omega_{v}$ denotes the valid pixel domain of the *v*-th view, and $c_{v,u}$ is the RGB color associated with the 3D point. The representation G can be used for geometry inspection, view consistency analysis, and visualization. In this work, G is not used merely for 3D reconstruction. Instead, it serves as the spatial carrier for projecting part-level visual–semantic consistency scores from 2D sensor views to the 3D prototype surface.

To ensure that identity-critical details are sufficiently observed, the sensing protocol includes a set of predefined views:

(7) $$V=\left\{V_{\mathrm{front}},V_{\mathrm{left}},V_{\mathrm{right}},V_{\mathrm{back}},V_{\mathrm{top}},V_{\mathrm{local}}\right\}.$$

The local close-up view $V_{\mathrm{local}}$ is optional and is used when small but important design elements, such as logos, facial features, or decorative patterns, need to be inspected.

### 2.3. Part-Aware Visual–Language Representation

A key idea of PartSense-IP is to evaluate IP consistency at the semantic part level. Let the concept image $I_{c}$ be decomposed into $K_{c}$ design-relevant regions:

(8) $$P_{c}=\left\{p_{c,1},p_{c,2},\ldots ,p_{c,K_{c}}\right\}.$$

Similarly, the prototype image captured from the *v*-th view is decomposed into

(9) $$P_{v}=\left\{p_{v,1},p_{v,2},\ldots ,p_{v,K_{v}}\right\}.$$

In practice, these regions can be obtained through a promptable segmentation model, manual annotation, or a hybrid procedure. The concept image provides the reference design parts, while the prototype views provide sensor-captured candidate parts. Each part is associated with a mask, a visual feature vector, a color descriptor, and an optional semantic label.

For each concept part $p_{c,k}$, a dense visual feature is extracted:

(10) $$f_{c,k}^{\mathrm{vis}}=\Psi_{\mathrm{vis}}\left(I_{c},p_{c,k}\right),$$

where $\Psi_{\mathrm{vis}}\left(\cdot \right)$ denotes the visual feature extractor. For each prototype part $p_{v,j}$, the corresponding feature is

(11) $$f_{v,j}^{\mathrm{vis}}=\Psi_{\mathrm{vis}}\left(I_{v},p_{v,j}\right).$$

The part-level visual similarity between a concept part $p_{c,k}$ and a prototype part $p_{v,j}$ is computed as

(12) $$s_{v,k,j}^{\mathrm{vis}}=\frac{{\left(f_{c,k}^{\mathrm{vis}}\right)}^{T}f_{v,j}^{\mathrm{vis}}}{{∥f_{c,k}^{\mathrm{vis}}∥}_{2}{∥f_{v,j}^{\mathrm{vis}}∥}_{2}}.$$

For each concept part, the best matched prototype part in view *v* is selected as

(13) $$j*\left(v,k\right)=\arg\max_{j}s_{v,k,j}^{\mathrm{vis}}.$$

Equation (13) provides an initial candidate correspondence. To avoid forcing every concept part to an arbitrary prototype region, the initial matches are subsequently refined using similarity, mask-overlap, semantic label, and view-visibility constraints. Specifically, a candidate match is accepted only when its normalized visual–semantic matching confidence exceeds a threshold $\tau_{\mathrm{match}}$ selected on the validation split. If no candidate satisfies this condition, the concept part is assigned to a null correspondence rather than being matched to the highest-scoring but unreliable region.

Duplicate assignments are resolved by enforcing that one prototype candidate can be assigned to at most one concept part within the same view. When two concept parts initially select the same candidate, the correspondence with the larger combined visual–semantic similarity is retained, while the remaining concept part is reassigned to its next-highest valid candidate. If no alternative candidate satisfies $\tau_{\mathrm{match}}$, that part is assigned to the null correspondence. This conflict-resolution procedure prevents the same prototype region from being repeatedly counted.

Missing parts and occluded parts are distinguished using the expected view visibility and cross-view observations. A part that is not expected to be visible from view *v*, or whose projected region is substantially occluded, is marked as ‘unobserved’ and is excluded from the normalization of the corresponding view-level score. By contrast, a part is regarded as potentially missing when it should be visible from one or more views but no valid candidate is found in those views. The null correspondence of a visible part is assigned a low consistency score and therefore contributes to missing-part detection.

Multiple-to-one cases may occur when a coarse candidate mask covers several neighboring concept parts. Such candidates are first refined using the associated local textual prompts and the dense feature boundaries. If the region can be reliably separated, the refined subregions are matched independently. Otherwise, the coarse region is assigned only to the highest-confidence concept part, while the remaining correspondences are marked as ambiguous and are not counted repeatedly. These rules provide a conservative treatment of uncertain correspondences and reduce false inconsistency scores caused by segmentation granularity or partial visibility.

The resulting part-level visual consistency score is

(14) $$S_{v,k}^{\mathrm{part}}=s_{v,k,j*\left(v,k\right)}^{\mathrm{vis}}.$$

For a valid correspondence, Equation (14) uses the similarity of the conflict-resolved candidate. We introduce a binary evaluability indicator $o_{v,k}\in \left\{0,1\right\}$ for the *k*-th concept part in view *v*. Here, $o_{v,k}=1$ indicates that the part is expected to be visible and can be evaluated from the current view, whereas $o_{v,k}=0$ indicates that the part is outside the visible region or is substantially occluded. A visible part assigned to the null correspondence remains evaluable, i.e., $o_{v,k}=1$, and its component consistency scores are set to zero because the absence of a valid candidate provides evidence of a missing part. In contrast, an unobserved part with $o_{v,k}=0$ is not assigned a zero score and is excluded from both view-level aggregation and cross-view stability computation.

#### 2.3.1. Cross-Domain Matching Considerations

The dense visual similarity in Equations (12)–(14) should not be interpreted as assuming exact appearance equivalence or complete domain invariance between a stylized 2D concept and a sensor-captured physical prototype. Differences in outlines, shading, perspective, material appearance, self-occlusion, and illumination may introduce noise into the dense visual correspondence [50,51]. PartSense-IP reduces the influence of such noise in several ways. First, the concept and prototype observations are independently decomposed into semantic parts, so that matching is performed between design-related regions rather than unrestricted whole-image patches. Second, candidate correspondences are verified using visual–semantic confidence, local textual prompts, mask compatibility, and view visibility; low-confidence candidates are rejected through the null assignment mechanism. Third, the DINOv2-based visual similarity is only one component of the final score and is jointly evaluated with view-normalized shape, color, vision–language semantic consistency, and cross-view evidence. Nevertheless, the current framework does not include an explicitly trained domain-adaptation or style-normalization module. Therefore, extreme differences between highly stylized concept artwork and photorealistic prototype observations may still produce uncertain local correspondences. This limitation is further examined through the independently captured real-prototype evaluation.

In addition to visual features, color and shape descriptors are extracted for each part. The color consistency between $p_{c,k}$ and its matched prototype part is defined as

(15) $$S_{v,k}^{\mathrm{color}}=1-d_{\mathrm{hist}}\left(h_{c,k},h_{v,j*\left(v,k\right)}\right),$$

where $h_{c,k}$ and $h_{v,j*\left(v,k\right)}$ are color histograms, and $d_{\mathrm{hist}}\left(\cdot \right)$ denotes a normalized histogram distance. The shape or silhouette consistency is defined as

(16) $$S_{v,k}^{\mathrm{shape}}=\mathrm{IoU}\left(M_{c,k},W_{v\to c}\left(M_{v,j*\left(v,k\right)}\right)\right),$$

where $M_{c,k}$ is the concept part mask, $M_{v,j*\left(v,k\right)}$ is the matched prototype part mask, and $W_{v\to c}\left(\cdot \right)$ denotes the view-normalization or alignment operation [52,53].

#### 2.3.2. Vision–Language Semantic Alignment

Visual similarity alone is insufficient for IP prototype evaluation because two regions may look similar at the pixel level but represent different design semantics. Therefore, PartSense-IP further introduces vision–language semantic alignment.

The concept-level semantic embedding is obtained from the concept image and optional text description:

(17) $$z_{c}^{\mathrm{sem}}=\Psi_{\mathrm{vl}}\left(I_{c},T_{c}\right),$$

where $\Psi_{\mathrm{vl}}\left(\cdot \right)$ denotes a vision–language encoder. For the sensor-captured prototype view $I_{v}$, the corresponding semantic embedding is

(18) $$z_{v}^{\mathrm{sem}}=\Psi_{\mathrm{vl}}\left(I_{v},T_{c}\right).$$

The view-level semantic consistency is then computed by cosine similarity:

(19) $$S_{v}^{\mathrm{sem}}=\frac{{\left(z_{c}^{\mathrm{sem}}\right)}^{T}z_{v}^{\mathrm{sem}}}{{∥z_{c}^{\mathrm{sem}}∥}_{2}{∥z_{v}^{\mathrm{sem}}∥}_{2}}.$$

This score evaluates whether the prototype view remains semantically aligned with the original concept design. For example, if the concept description emphasizes a rounded toy-like style, a specific symbolic logo, or a cute mascot identity, the semantic alignment module checks whether these design semantics are still recognizable in the captured prototype views.

To further capture part-level semantics, each design-relevant part can also be associated with a local textual prompt $T_{c,k}$, such as “round ear”, “star-shaped logo”, or “blue glossy body”. The part-level semantic consistency is defined as

(20) $$S_{v,k}^{\mathrm{sem}}=\frac{{\left(z_{c,k}^{\mathrm{sem}}\right)}^{T}z_{v,j*\left(v,k\right)}^{\mathrm{sem}}}{{∥z_{c,k}^{\mathrm{sem}}∥}_{2}{∥z_{v,j*\left(v,k\right)}^{\mathrm{sem}}∥}_{2}}.$$

The concrete implementation of part extraction, semantic labeling, correspondence assignment, and missing-part handling is specified in Section 2.4.

### 2.4. Reproducible Part Extraction and Correspondence Protocol

Candidate part masks are generated using the frozen SAM ViT-H checkpoint sam_vit_h_4b8939.pth, which are elaborated upon in Section 4.2. Automatic mask generation is performed within the detected object bounding box using a 32×32 point grid. Masks with a predicted-IoU score below 0.88, a stability score below 0.92, or an area smaller than 0.5% of the foreground-object area are discarded. For strongly overlapping masks with an IoU greater than 0.70, only the mask with the larger predicted-IoU score is retained.

For each concept, the local part vocabulary is constructed once from the noun or adjective–noun phrases explicitly appearing in the design description $T_{c}$, supplemented by the fixed generic vocabulary {*head, body, eye, ear, mouth, nose, limb, logo, accessory, decoration, surface pattern*}. No language model is used to generate or modify these labels at test time. Each concept mask is assigned the label whose CLIP text embedding has the highest cosine similarity with the masked visual embedding. The corresponding local prompt $T_{c,k}$ and semantic label are then fixed and reused for all prototype variants and sensing views of the same concept. A mask whose maximum semantic similarity is below 0.25 is retained as an unlabeled candidate and is matched using the visual and geometric terms only.

For concept part $p_{c,k}$ and candidate prototype part $p_{v,j}$, the normalized matching confidence is defined as

(21) $$c_{v,k,j}=0.50\;{\tilde{s}}_{v,k,j}^{\mathrm{vis}}+0.30\;{\tilde{s}}_{v,k,j}^{\mathrm{sem}}+0.20\;s_{v,k,j}^{\mathrm{mask}},$$

where ${\tilde{s}}_{v,k,j}^{\mathrm{vis}}$ and ${\tilde{s}}_{v,k,j}^{\mathrm{sem}}$ denote the visual and semantic cosine similarities normalized to [0,1], respectively, and 

(22) $$s_{v,k,j}^{\mathrm{mask}}=\mathrm{IoU}\left(M_{c,k},W_{v\to c}\left(M_{v,j}\right)\right)$$

measures mask compatibility after view normalization. The coefficients in (21) and the acceptance threshold $\tau_{\mathrm{match}}=0.60$ are determined using only the validation subset and remain fixed during testing. A correspondence is accepted only when $c_{v,k,j}\geq \tau_{\mathrm{match}}$. Candidate correspondences are sorted in descending order of $c_{v,k,j}$ and assigned greedily under a one-to-one constraint within each view. Therefore, one prototype candidate cannot be assigned to multiple concept parts. If the highest-confidence candidate has already been assigned, the next valid candidate is considered; if no remaining candidate satisfies $\tau_{\mathrm{match}}$, a null correspondence is retained.

Cross-view association is anchored by the concept-part index *k* rather than by unconstrained prototype-to-prototype matching. Thus, candidates matched to the same concept part in different views are treated as observations of the same semantic component. Let ${\hat{M}}_{v,k}$ denote the expected region of concept part *k* in view *v* after pose-based view normalization. The part is considered evaluable when at least 50% of ${\hat{M}}_{v,k}$ lies inside the prototype foreground and at least 60% of this region contains valid depth measurements. A projected point is treated as occluded when it lies more than 20 mm behind the observed depth surface; if more than 50% of the expected region is occluded, the part is marked as unobserved and $o_{v,k}=0$.

When the part satisfies the above visibility conditions but no candidate mask reaches $\tau_{\mathrm{match}}$, it is assigned a null correspondence, $o_{v,k}=1$, and its shape, color, visual, and semantic consistency scores are set to zero. A part is declared potentially missing at the prototype level when no valid correspondence is found in any of its evaluable views. In contrast, non-visible or substantially occluded parts are excluded from both view-level aggregation and cross-view stability computation.

## 3. Part-Aware Consistency Fusion and Feedback

This section presents the core Part-Aware Visual–Semantic Consistency Fusion (PVCF) algorithm and the feedback-generation mechanism of PartSense-IP. Based on the multi-view sensing representation and part-level visual–language features obtained in Section 2, the proposed algorithm produces a unified IP consistency score, localizes inconsistent regions, and generates interpretable feedback for prototype refinement.

### 3.1. Part-Aware Visual–Semantic Consistency Fusion (PVCF)

The core algorithm of PartSense-IP is the Part-Aware Visual–Semantic Consistency Fusion (PVCF) algorithm, which integrates shape, color, part-level visual appearance, semantic alignment, and multi-view stability into a unified IP consistency score. PVCF is not introduced as a new visual feature extractor or foundation model. Instead, it serves as a structured decision layer that converts heterogeneous pretrained representations into design-oriented consistency measurements. A direct concatenation or averaging of the backbone outputs would obscure the different meanings of shape, color, local appearance, and semantic deviations and would not reveal which design attribute causes an inconsistency. PVCF therefore retains these measurements as explicit component scores and aggregates them hierarchically over matched parts and sensing views.

For the *k*-th concept part observed from the *v*-th view, the part-level consistency score is defined as

(23) $$S_{v,k}=\alpha_{1}S_{v,k}^{\mathrm{shape}}+\alpha_{2}S_{v,k}^{\mathrm{color}}+\alpha_{3}S_{v,k}^{\mathrm{part}}+\alpha_{4}S_{v,k}^{\mathrm{sem}},$$

where $\alpha_{1}$, $\alpha_{2}$, $\alpha_{3}$, and $\alpha_{4}$ are non-negative weighting factors satisfying $\sum_{i=1}^{4}\alpha_{i}=1$. Since different IP components contribute differently to the visual identity of a concept, each concept part is assigned an identity weight $\omega_{k}$. In the current implementation, $\omega_{k}$ is not learned from the prototype observations or evaluation labels. Instead, it is assigned using the following fixed three-level concept-side rubric:

$$\omega_{k}=\left\{\begin{array}{cc} 3, & \mathrm{identity-defining}\;\mathrm{part},\\ 2, & \mathrm{major}\;\mathrm{structural}\;\mathrm{or}\;\mathrm{appearance}\;\mathrm{part},\\ 1, & \mathrm{secondary}\;\mathrm{or}\;\mathrm{supporting}\;\mathrm{part}. \end{array}\right.$$

Identity-defining parts include logos, facial features, symbolic accessories, and characteristic decorations explicitly identified by the concept description or local part prompt. Major parts include the head, body, limbs, dominant silhouette, and principal color-bearing regions. Other non-distinctive regions are assigned the secondary level. When no part-specific identity cue is available, uniform weights $\omega_{k}=1$ are used. Because Equations (24) and (26) normalize the weights over the valid parts, these raw values are used directly without additional global normalization. The consistency score of the *v*-th view is computed as

(24) $$S_{v}=\frac{\sum_{k=1}^{K_{c}}o_{v,k}\omega_{k}S_{v,k}}{\sum_{k=1}^{K_{c}}o_{v,k}\omega_{k}},$$

where $o_{v,k}$ is the evaluability indicator defined above. Therefore, the identity weights are renormalized over only the parts that are evaluable in view *v*. A part that should be visible but is assigned to the null correspondence remains in Equation (24) with a zero consistency score, while a genuinely unobserved part is excluded through $o_{v,k}=0$.

It is important to distinguish the roles of the component-level coefficients ${\left\{\alpha_{i}\right\}}_{i=1}^{4}$ and the part-identity weights ${\left\{\omega_{k}\right\}}_{k=1}^{K_{c}}$. The coefficients $\alpha_{i}$ specify the benchmark-level balance among shape, color, dense visual, and semantic evidence and are shared across all samples. In contrast, $\omega_{k}$ is a concept-specific but non-learned weight assigned using the fixed three-level rubric above. Its assignment depends only on the concept-side part label, textual design description, and fixed local part prompt, and does not use the prototype prediction, ground-truth inconsistency mask, or test-set result. The same rule is applied to all training, validation, and test samples. Nevertheless, the current implementation uses a common set of component-level coefficients ${\left\{\alpha_{i}\right\}}_{i=1}^{4}$ for all test samples. This choice avoids sample-specific test-time fitting and preserves a transparent and reproducible scoring rule, but it does not constitute fully adaptive product-level weighting among shape, color, visual, and semantic evidence. A natural extension is to infer sample-dependent component coefficients from the concept description, part attributes, or a lightweight gating network, subject to non-negativity and normalization constraints.

To account for multi-view stability, PartSense-IP further evaluates whether the same design part remains consistent across different views. Let $V_{k}=\left\{v|o_{v,k}=1\right\}$ denote the set of evaluable views for part *k*, and let $q_{k}=I(|V_{k}\left|\geq 2\right)$, we have

(25) $$S_{k}^{\mathrm{view}}=1-\mathrm{Var}\left({\left\{S_{v,k}\right\}}_{v\in V_{k}}\right),\;\left|V_{k}\right|\geq 2,$$

where Var(·) denotes the normalized variance. Thus, cross-view variance is computed only over views in which the part is expected to be visible and evaluable. Unobserved or heavily occluded views are not replaced by zero values. When a part is evaluable in fewer than two views, its cross-view stability cannot be reliably estimated, and the part is excluded from the stability term in the final fusion.

The final IP consistency score is obtained as

(26) $$S_{\mathrm{IP}}=\beta_{1}\sum_{v=1}^{N}\rho_{v}S_{v}+\beta_{2}\frac{\sum_{k=1}^{K_{c}}q_{k}\omega_{k}S_{k}^{\mathrm{view}}}{\sum_{k=1}^{K_{c}}q_{k}\omega_{k}},$$

where $q_{k}$ excludes parts for which fewer than two evaluable views are available. The identity weights in the cross-view term are therefore renormalized over only the parts with sufficient visibility. If a complete sensing view contains no evaluable part, that view is omitted and the remaining view weights $\rho_{v}$ are renormalized accordingly. The score $S_{\mathrm{IP}}$ provides a quantitative measurement of how well the prototype preserves the original IP design. However, a single scalar score is insufficient for design refinement. Therefore, PartSense-IP also generates a difference map by projecting part-level inconsistency scores back to the sensor views:

(27) $$M_{\mathrm{diff}}\left(u,v\right)=1-S_{v,k},\;u\in M_{v,j*\left(v,k\right)}.$$

Regions with larger values in $M_{\mathrm{diff}}$ indicate stronger visual–semantic deviations from the original concept design.

The localized inconsistency scores can also be projected onto the 3D prototype representation G, producing a 3D inconsistency map:

(28) $$M_{3D}\left(X_{v,u}\right)=M_{\mathrm{diff}}\left(u,v\right).$$

This allows designers to inspect inconsistent regions directly on the 3D prototype surface.

### 3.2. Difference Localization and Interpretable Feedback

In addition to quantitative scoring and localization, PartSense-IP produces interpretable feedback for design refinement. For each part $p_{c,k}$, the framework checks which consistency component contributes most to the inconsistency:

(29) $$r_{v,k}=\arg\min\left\{S_{v,k}^{\mathrm{shape}},S_{v,k}^{\mathrm{color}},S_{v,k}^{\mathrm{part}},S_{v,k}^{\mathrm{sem}}\right\}.$$

The result $r_{v,k}$ indicates whether the inconsistency is mainly caused by shape distortion, color deviation, local visual mismatch, or semantic identity loss.

Based on this diagnosis, a structured feedback report is generated:

(30) $$F_{\mathrm{fb}}=R\left(S_{\mathrm{IP}},M_{\mathrm{diff}},\left\{r_{v,k}\right\}\right),$$

where R(·) denotes a rule-based or language-model-assisted report generator. Example feedback includes: “the side-view silhouette of the ears deviates from the original concept”, “the logo region is partially missing under oblique views”, or “the dominant color of the prototype body is inconsistent with the concept artwork.” The feedback module is designed to support practical design iteration. Instead of only telling designers whether a prototype is good or bad, it explains where the inconsistency occurs and what type of design attribute is affected. This makes the proposed framework suitable for IP product review, game-style asset prototyping, cultural creative product inspection, and design education.

The complete PartSense-IP procedure is summarized in Algorithm 1. The algorithm takes the concept design and multi-view RGB-D sensor observations as inputs and outputs the IP consistency score, localized difference map, and interpretable feedback.
**Algorithm 1** PartSense-IP  framework**Require:** Concept design $C=\left\{I_{c},T_{c}\right\}$; multi-view RGB-D observations $S={\left\{I_{v},D_{v},P_{v}\right\}}_{v=1}^{N}$ **Ensure:** IP consistency score $S_{\mathrm{IP}}$; difference map $M_{\mathrm{diff}}$; feedback report $F_{\mathrm{fb}}$
 1:Capture the prototype from multiple identity-critical and geometry-critical views. 2:Back-project RGB-D observations and construct the multi-view prototype representation G. 3:Segment the concept image into design-relevant parts $P_{c}$. 4:**for** each view v=1,…,N **do** 5:    Segment the prototype view into candidate parts $P_{v}$. 6:    **for** each concept part $p_{c,k}$ **do** 7:      Extract visual, color, shape, and semantic features. 8:      Determine the evaluability indicator $o_{v,k}$ using the expected view visibility and occlusion status. 9:      If $o_{v,k}=1$, perform conflict-resolved part matching; retain a null correspondence with zero consistency when no valid candidate is found.10:     Compute the component consistency scores for evaluable parts and exclude unobserved parts from the view-level normalization.11:     Fuse the component scores to obtain $S_{v,k}$.12:   **end for**13:   Compute the view-level consistency score $S_{v}$.14:**end for**15:Compute $S_{k}^{\mathrm{view}}$ only over the evaluable view set $V_{k}=\left\{v|o_{v,k}=1\right\}$; exclude parts with fewer than two evaluable views from the stability term.16:Fuse view-level and part-level consistency scores to obtain $S_{\mathrm{IP}}$.17:Generate the localized difference map $M_{\mathrm{diff}}$ and project it onto G if needed.18:Generate interpretable design feedback $F_{\mathrm{fb}}$ according to the dominant inconsistency sources.19:**return** $S_{\mathrm{IP}}$, $M_{\mathrm{diff}}$, $F_{\mathrm{fb}}$


## 4. Experimental Design and Evaluation Protocol

### 4.1. Dataset Construction and Multi-View Sensing Setup

To evaluate the proposed PartSense-IP framework, we construct a task-specific dataset, termed *IP-ProtoSense*, for sensor-captured visual–semantic consistency evaluation of IP prototypes. Since existing public datasets are not directly designed for evaluating the consistency between 2D IP concept designs and physical or digital product prototypes, the proposed dataset is built by combining publicly available 3D object assets, controlled prototype-variant generation, multi-view RGB-D rendering, and a small real-captured prototype subset. This design allows us to obtain reliable ground-truth annotations while maintaining a practical connection to real sensing scenarios.

Each sample in the dataset is organized as

(31) $$D_{i}=\left\{I_{c}^{i},T_{c}^{i},{\left\{I_{v}^{i},D_{v}^{i},P_{v}^{i}\right\}}_{v=1}^{N},Y^{i},M_{\mathrm{gt}}^{i}\right\},$$

where $I_{c}^{i}$ is the reference concept image, $T_{c}^{i}$ is the textual design description, $I_{v}^{i}$ is the RGB observation captured or rendered from the *v*-th view, $D_{v}^{i}$ is the corresponding depth map, $P_{v}^{i}$ is the camera pose or view metadata, $Y^{i}$ is the human-annotated overall consistency score, and $M_{\mathrm{gt}}^{i}$ denotes the ground-truth inconsistency mask when local deviations are available.

The dataset is constructed through the following three-stage procedure.

**Stage 1: Source asset collection and concept-reference construction.** We first collect candidate 3D assets from publicly available object repositories, including toy-like, character-like, cultural creative, and product-like objects from Objaverse [46], Amazon Berkeley Objects [47], Google Scanned Objects [48], and OmniObject3D [49]. These repositories provide a diverse source of textured 3D objects and are suitable for constructing multi-view sensing benchmarks. To avoid copyright and commercial IP issues, assets that clearly correspond to protected commercial characters or brands are excluded. We only retain generic, publicly licensed, or self-designed stylized objects.

For each selected base asset, the reference concept image $I_{c}$ is constructed according to its source. Among the 300 source assets, 240 are obtained from public 3D repositories, while 60 correspond to self-designed concept–prototype pairs. For the self-designed subset, $I_{c}$ is the original independently created 2D concept artwork. For the public-asset subset, $I_{c}$ is generated as a canonical stylized front-view rendering of the corresponding clean 3D asset. A textual description $T_{c}$ is then manually written to summarize the identity-critical attributes of each concept, including its category, dominant colors, symbolic elements, local decorations, material style, and intended visual semantics. The two subsets represent different levels of evaluation difficulty. The self-designed subset involves an independently created 2D concept and therefore contains a larger appearance and representation gap between the concept and prototype. In contrast, the public-asset subset shares the underlying geometry and texture source between the canonical concept rendering and the clean prototype. This same-source construction reduces the concept-to-prototype domain gap and is intentionally retained to support controlled, spatially traceable evaluation of isolated deviations.

Accordingly, results on the public-asset subset should be interpreted as diagnostic evidence for inconsistency scoring and localization, rather than as validation of a fully independent design-to-prototype realization process. We therefore additionally report performance separately for the public-asset and self-designed subsets and use the independently captured real-prototype subset to examine transfer beyond the controlled rendering pipeline.

**Stage 2: Prototype-variant generation with controlled design deviations.** For each clean concept–prototype pair, we generate multiple prototype variants to simulate common design-review problems. The clean variant is treated as the visually consistent prototype, while inconsistent variants are generated by modifying geometry, color, texture, local components, or semantic identity. Specifically, we consider six types of design deviations:

(32) $$E=\left\{E_{\mathrm{color}},E_{\mathrm{shape}},E_{\mathrm{missing}},E_{\mathrm{disp}},E_{\mathrm{texture}},E_{\mathrm{semantic}}\right\},$$

where $E_{\mathrm{color}}$ denotes dominant color deviation, $E_{\mathrm{shape}}$ denotes shape distortion, $E_{\mathrm{missing}}$ denotes missing symbolic elements such as logos or accessories, $E_{\mathrm{disp}}$ denotes part displacement, $E_{\mathrm{texture}}$ denotes texture or material inconsistency, and $E_{\mathrm{semantic}}$ denotes visual–semantic identity loss.

The controlled deviations are not intended to represent the full complexity of industrial fabrication errors [54,55]. Their primary purpose is to provide isolated and spatially traceable test cases for measuring whether an evaluation method responds to specific design attributes. The six categories were selected to cover common prototype-review dimensions, including geometry, component completeness and placement, color, material appearance, and semantic identity. In practical prototypes, these deviations may occur simultaneously and may not have sharply defined boundaries. We therefore treat the controlled subset as a diagnostic benchmark rather than as a complete substitute for real-world validation.

**Stage 3: Multi-view RGB-D sensing and rendering.** Each prototype variant is observed from multiple predefined viewpoints to simulate a practical RGB-D sensing process. The view set is defined as

(33) $$V=\left\{V_{\mathrm{front}},V_{\mathrm{left}},V_{\mathrm{right}},V_{\mathrm{back}},V_{\mathrm{top}},V_{\mathrm{local}}\right\}.$$

The front view is mainly used to capture the primary identity of the IP product, the side and back views are used to inspect geometry and cross-view consistency, and the top and local close-up views are used to observe fine-grained structures such as logos, facial features, and decorative components.

The real-captured subset was acquired using an Intel RealSense D455 RGB-D camera. The RGB stream was recorded at a resolution of 1280×720, while the depth stream was recorded at 848×480, both at 30 frames per second. During acquisition, each prototype was placed approximately 0.6–1.2 m from the camera. This range ensured that the complete prototype remained inside the effective depth range while small identity-critical regions, such as facial features, logos, and decorative components, retained sufficient spatial resolution.

The factory-provided intrinsic calibration parameters were used for the RGB and depth cameras. Depth-to-color registration was performed using the Intel RealSense SDK, and the registered depth maps were subsequently used for RGB-D back-projection. Invalid depth values and isolated outliers were removed before reconstruction. A 5×5 median filter was applied to suppress impulsive depth noise, followed by a bilateral filter to reduce local depth fluctuations while preserving object boundaries. The samples were captured under stable indoor diffuse illumination using two fixed LED panels positioned on the left and right sides of the camera. Direct reflections and severe cast shadows were avoided where possible. The illumination arrangement remained unchanged for the standard real-captured evaluation, while lighting variations were introduced separately only in the robustness experiments.

The front, left, right, back, top, and local close-up views were acquired using a calibrated turntable. The camera remained fixed, and the turntable rotation angle provided the initial relative pose for each view. The initial poses were further refined through point-to-plane iterative closest point registration between adjacent RGB-D point clouds. Samples exhibiting severe missing depth, failed registration, or insufficient object coverage were reacquired. The same acquisition, registration, and depth-filtering procedure was applied to all real-captured samples.

For the real-captured subset, physical toys, cultural creative products, and 3D-printed prototypes are captured using a low-cost multi-view imaging setup. Unlike the controlled synthetic subset, the real samples are not generated by applying one isolated rendering operator. They contain naturally coupled variations arising from physical fabrication, assembly, material appearance, surface finish, decorative completeness, and sensing conditions. Consequently, a real sample may exhibit several simultaneous deviations, such as a local geometric mismatch together with a color or texture difference. The 60 real-captured samples are treated as an independent challenge subset. They are excluded from model configuration, PVCF coefficient selection, threshold determination, and all other validation procedures. All settings selected on the synthetic training and validation subsets are directly applied to the real-captured samples without further adjustment. This protocol is used to assess synthetic-to-real generalization and to reduce the possibility that the evaluation favors the controlled deviation operators used in dataset construction.

To make the dataset scale explicit, Table 1 summarizes the statistics of IP-ProtoSense. In the current implementation, we collect 300 generic or self-designed source assets and construct one concept reference for each asset. For each concept–prototype pair, one clean prototype and six controlled inconsistent variants are generated, corresponding to color shift, shape distortion, missing symbolic element, part displacement, texture inconsistency, and semantic identity loss. Each prototype variant is observed from six predefined views, including front, left, right, back, top, and local close-up views. This results in 2100 prototype samples and 12,600 multi-view RGB-D observations in the synthetic subset. In addition, 60 real-captured prototype samples are collected to evaluate the applicability of the proposed framework under practical sensing conditions.

Regarding the annotation protocol, three annotators independently evaluated each concept–prototype pair. The annotator group included one annotator with industrial/product-design experience, one annotator with 3D modeling and prototype-development experience, and one annotator with computer-vision and visual-inspection experience. Before formal annotation, all annotators completed a calibration session using a separate set of examples that was not included in the final evaluation data. The calibration session was used to clarify the rating criteria and the treatment of ambiguous or partially visible inconsistencies.

For each sample, the annotators were provided with the concept image, the textual design description, and all available multi-view prototype observations. Each annotator independently provided: (1) an overall design-consistency rating; (2) the primary inconsistency category and affected part, when applicable; and (3) a binary mask for each visually identifiable inconsistent region.

The overall consistency rating was assigned using the following five-point scale:

- 5: Highly consistent, with no visible identity-critical deviation;
- 4: Generally consistent, with only minor deviations that do not substantially affect the intended identity;
- 3: Moderately consistent, with noticeable local deviations but with the main design identity preserved;
- 2: Weakly consistent, with major deviations in one or more identity-critical attributes;
- 1: Clearly inconsistent, with substantial loss of the intended visual or semantic identity.

The five-point scores were linearly normalized to [0,1] using Y=(r−1)/4, where r∈{1,2,3,4,5}. The final sample-level human rating was obtained by averaging the independently assigned normalized scores of the three annotators.

For inconsistency localization, annotators only marked regions that could be visually attributed to a mismatch with the concept reference. Lighting artifacts, shadows, background regions, and sensor noise were not labeled unless they obscured or altered an identity-critical design attribute. The initial masks were retained for agreement analysis. After agreement statistics were computed, disagreements were reviewed jointly, and a consensus mask was produced for the final localization evaluation.

Regarding the inter-rater agreement, the reliability of the human annotations was quantified before consensus resolution. For the continuous overall consistency ratings, we report the two-way random-effects, absolute-agreement intraclass correlation coefficient based on the average of the three annotators, ICC (2, *k*). Fleiss’ κ is used to evaluate agreement on the categorical inconsistency labels, while localization agreement is measured by the mean pairwise Dice coefficient among the three original binary masks. These metrics evaluate the reliability of the human reference independently of the predictions produced by the evaluated methods.

As reported in Table 2, the overall consistency ratings achieve an ICC (2, *k*) of 0.886, indicating strong agreement among the three annotators. The Fleiss’ κ of 0.817 for the inconsistency categories also shows that the annotators generally agreed on the dominant type of design deviation. The mean pairwise Dice coefficient of 0.842 indicates reliable spatial agreement for the local inconsistency masks, although localization naturally exhibits slightly greater ambiguity near deviation boundaries. These results support the use of the averaged human ratings and consensus masks as reference labels for consistency scoring and localization evaluation.

Annotators were further instructed to distinguish genuine design inconsistency from the expected appearance gap between a stylized 2D concept and a sensor-captured physical prototype. Differences caused solely by viewpoint, perspective, illumination, shading, camera response, surface reflectance, material rendering, or minor manufacturing tolerance were not labeled as inconsistencies unless they altered an identity-critical shape, color, symbolic element, local appearance, or semantic attribute. For real-captured prototypes, the transition between consistent and inconsistent regions may be gradual rather than strictly binary. In such cases, the annotators marked the principal perceptually affected region according to the intended design attribute, rather than attempting to define an exact physical boundary between modified and unmodified material.

### 4.2. Baselines and Implementation Details

We compared the proposed PartSense-IP framework with a few representative baselines. These baselines were selected from recent and widely used vision–language, dense visual representation, segmentation, and 3D multimodal learning methods. They were adapted to the IP prototype consistency evaluation task to provide a fair comparison.

- **CLIP-based global visual–semantic matching [19].** This baseline employs CLIP to compute global image–text and image–image similarity. For each prototype, the RGB images captured from different views are encoded by the CLIP image encoder, while the textual design description is encoded by the CLIP text encoder. The final consistency score is obtained by averaging the cosine similarities across all views. This baseline evaluates whether a global vision–language representation can measure the semantic consistency between the concept design and the sensor-captured prototype. However, it does not explicitly model part-level consistency, depth information, or multi-view geometric stability.
- **DINOv2-based dense visual feature matching [25].** This baseline uses DINOv2 to extract robust dense visual features from the concept image and prototype views. The similarity between the concept artwork and each prototype view is computed based on feature-level cosine similarity. The final score is obtained by averaging the view-level similarities. This baseline provides a strong visual representation method for appearance-level comparison. Nevertheless, it mainly focuses on visual feature similarity and does not incorporate textual design semantics or sensor-based 3D fusion.
- **SAM-assisted part-level CLIP matching [19,28].** This combines the Segment Anything Model (SAM) with CLIP-based semantic matching. SAM is first used to segment the concept image and prototype views into candidate object parts. For each concept part, the most similar prototype part is selected according to CLIP visual feature similarity. The overall consistency score is then computed by averaging the matched part-level similarities. This baseline introduces part-level comparison and can detect some local inconsistencies. However, it operates only on 2D RGB images and does not use depth sensing, view-pose information, or cross-view stability modeling.
- **ULIP-based 3D multimodal matching [38].** This baseline uses ULIP, which learns a unified representation space for language, images, and point clouds. For each prototype, the multi-view RGB-D observations are fused into a point-cloud representation. The point-cloud feature is then compared with the concept image and textual description in the shared multimodal feature space. This baseline is included because it represents a recent 3D multimodal learning strategy for aligning visual, textual, and 3D information. However, it does not explicitly evaluate IP-specific part consistency, local decorative elements, or view-dependent design deviations.
- **BLIP-2-based multimodal matching [21].** BLIP-2 is included as a stronger global vision–language baseline beyond CLIP. The concept reference, textual description, and prototype views are encoded using the frozen visual encoder and Q-Former. The normalized view-level multimodal similarities are averaged to obtain the final prototype consistency score.
- **Grounding DINO + CLIP [12,24,25,37].** Grounding DINO is used to localize identity-critical regions according to the same textual part descriptions used by the proposed framework. The grounded concept and prototype regions are subsequently compared using CLIP embeddings. The matched part similarities are averaged over parts and views. This baseline provides language-guided part-level comparison without RGB-D projection, identity-aware fusion, or cross-view stability modeling.
- **OpenShape-based 3D multimodal matching [41].** OpenShape is included as an additional 3D vision–language baseline beyond ULIP. Multi-view RGB-D observations are fused into a colored point cloud, which is encoded using the pretrained OpenShape model and compared with the concept-image and textual representations in the shared embedding space.
- **Part-CLIP**. This baseline extracts spatial patch tokens from the CLIP visual Transformer and performs local matching within the corresponding concept and prototype part masks. The part score is computed from the average cosine similarity of the matched patch features.
- **Part-DINOv2-MNN**. Part-DINOv2-MNN extracts the final-block DINOv2 patch tokens and constructs dense correspondences using mutual nearest-neighbor matching. Only reciprocal patch correspondences inside the matched part masks are retained, and their average cosine similarity is used as the part-level score.
- **Part-ULIP.** This scheme projects each visible 2D part mask into the fused RGB-D point cloud and encodes the resulting part-level point subset using the same pretrained ULIP backbone as the object-level baseline. The prototype score is obtained by uniformly averaging the matched part-level similarities across valid parts and views.
- **Proposed method:** Our proposed PartSense-IP employs the same input concept image, textual description, and multi-view RGB-D observations as the baselines. The proposed Part-Aware Visual–Semantic Consistency Fusion (PVCF) algorithm integrates shape consistency, color consistency, part-level visual similarity, semantic alignment, and cross-view stability. The final score is computed according to Section 3.1. In addition to the scalar consistency score, PartSense-IP also outputs localized inconsistency maps and interpretable design feedback.

All pretrained models are implemented using their publicly released repositories and checkpoints and are kept frozen during evaluation. Unless otherwise stated, RGB inputs are resized to 224×224 and normalized using the preprocessing procedure associated with the corresponding pretrained model. All similarity scores are computed using *ℓ*_2_-normalized features. Hyperparameters, prompt templates, segmentation thresholds, and point-cloud preprocessing parameters are selected using only the validation split and are then fixed for all test experiments.

For text-conditioned models, the global prompt is constructed from the complete design description, while local prompts correspond to identity-critical part descriptions, such as “round ear”, “star-shaped logo”, or “blue glossy body”. The same local prompts are used by PartSense-IP and Grounding DINO + CLIP. Prompt wording is fixed before test-set evaluation and is not optimized separately for individual samples. The detailed model variants, checkpoints, feature layers, and key settings are summarized in Table 3.

The implementation details are summarized in Table 4. All RGB images are resized to 224×224 before being fed into CLIP and DINOv2. Depth maps are normalized according to the valid depth range of each view and are used for view alignment, point-cloud construction, and 3D inconsistency projection. SAM is used to generate candidate part masks for both concept images and prototype views, and small isolated masks are removed using an area threshold. DINOv2 features are used as dense visual descriptors for local appearance comparison, while CLIP features are used for image–text and part–text semantic alignment. For ULIP, the multi-view RGB-D observations are fused into a point-cloud representation and compared with the concept image and textual description in the shared multimodal space.

The weighting factors of the proposed PVCF algorithm are selected on the validation set and then fixed for testing. Unless otherwise specified, we set $\alpha_{1}=0.25$, $\alpha_{2}=0.20$, $\alpha_{3}=0.30$, and $\alpha_{4}=0.25$ for shape, color, dense visual similarity, and semantic consistency, respectively. The view-level fusion parameters are set to $\beta_{1}=0.80$ and $\beta_{2}=0.20$, where the first term measures average multi-view consistency and the second term measures cross-view stability. For view weights, the front and local close-up views are assigned slightly larger weights because they usually contain more identity-critical information, while the remaining views are used to improve geometric and appearance stability.

All PVCF weights and thresholds are selected using only the synthetic validation split and remain fixed during testing. The component weights ${\left\{\alpha_{i}\right\}}_{i=1}^{4}$ are first optimized by constrained linear regression against the human consistency ratings, subject to $\alpha_{i}\geq 0$ and $\sum_{i}\alpha_{i}=1$. The resulting coefficients (0.24,0.18,0.32,0.26) are rounded to the fixed setting (0.25,0.20,0.30,0.25) for improved transparency and reproducibility. The view-stability weights are selected by grid search, resulting in $\left(\beta_{1},\beta_{2}\right)=\left(0.80,0.20\right)$. The part-matching threshold, sample-level detection threshold, and heatmap binarization threshold are selected by maximizing validation part-matching F1-score, detection F1-score, and localization mIoU, respectively. The resulting values are $\tau_{\mathrm{match}}=0.60$, $\tau_{\det}=0.75$, and $\tau_{\mathrm{loc}}=0.50$. A prototype is classified as inconsistent when $S_{\mathrm{IP}}<\tau_{\det}$. No controlled-test or real-captured sample is used for weight or threshold selection.

### 4.3. Performance Evaluation

**Figure 2:**
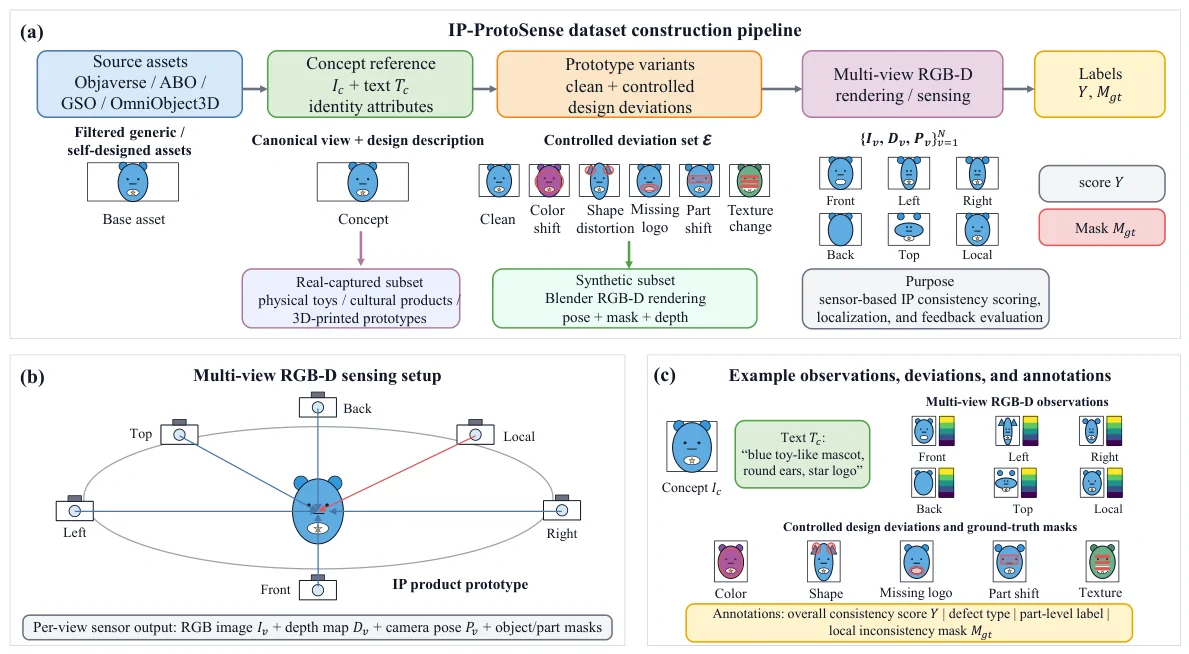
Dataset construction and multi-view sensing setup of IP-ProtoSense. (**a**) The dataset is constructed from public or self-designed source assets, concept references, controlled prototype variants, multi-view RGB-D observations, and annotation labels. Controlled deviations are introduced to simulate practical design-review problems, including color shift, shape distortion, missing symbolic elements, part displacement, and texture inconsistency. (**b**) The multi-view sensing setup captures the prototype from identity-critical and geometry-critical viewpoints, including front, side, back, top, and local close-up views. Each view provides an RGB image $I_{v}$, a depth map $D_{v}$, camera pose or view metadata $P_{v}$, and object/part masks. (**c**) Each sample contains the reference concept image, textual design description, multi-view RGB-D observations, ground-truth inconsistency masks, and annotation labels, supporting consistency scoring, inconsistency detection, localization, and interpretable design feedback.

Figure 2 illustrates the construction procedure of the proposed IP-ProtoSense dataset and the corresponding multi-view RGB-D sensing setup. As shown in Figure 2a, the dataset is constructed through a progressive pipeline from source assets to annotated sensing samples. First, candidate source assets are collected from public 3D object repositories or self-designed IP-style models. To avoid commercial copyright issues, only generic, publicly licensed, or self-designed toy-like, mascot-like, cultural creative, and product-like assets are retained. For each selected asset, a canonical front-view rendering is used as the reference concept image $I_{c}$, and a textual description $T_{c}$ is manually written to summarize its identity-critical design attributes, such as dominant color, object category, local decoration, symbolic logo, material style, and intended visual semantics. After obtaining the concept reference, multiple prototype variants are generated to simulate practical design-review scenarios. The clean variant is regarded as the visually consistent prototype, while inconsistent variants are generated by introducing controlled deviations, including color shift, shape distortion, missing symbolic elements, part displacement, texture inconsistency, and semantic identity change. These deviations correspond to common problems in IP product prototyping, where a prototype may preserve the overall appearance but lose important local identity cues, such as a logo, ear shape, decorative pattern, or intended toy-like style. Since the deviation operations are controlled during data generation, the inconsistent regions can be projected to each rendered view to obtain ground-truth local inconsistency masks $M_{\mathrm{gt}}$.

Figure 2b shows the multi-view RGB-D sensing configuration. The prototype is observed from identity-critical and geometry-critical views, including the front, left, right, back, top, and local close-up views. The front view mainly captures the most representative IP identity, while the side and back views provide complementary information for evaluating cross-view geometric and appearance consistency. The top view helps inspect global structural deformation, and the local close-up view is used to examine small but important design elements, such as logos, facial features, and decorative accessories. For each view, the sensing output contains an RGB image $I_{v}$, a depth map $D_{v}$, camera pose or view metadata $P_{v}$, and object/part masks. Therefore, each sample can be represented as a multi-view observation set ${I_{v},D_{v},P_{v}}_{v=1}^{N}$. Figure 2c gives an example of the data organization. Each sample contains the reference concept image, the textual design description, multi-view RGB-D observations, controlled deviation types, ground-truth inconsistency masks, and annotation labels. The annotations include an overall consistency score *Y*, part-level defect labels, and local inconsistency masks $M_{\mathrm{gt}}$. This organization allows the proposed framework to be evaluated from three perspectives: whether it can accurately predict the overall visual–semantic consistency, whether it can detect different types of prototype deviations, and whether it can localize the inconsistent regions that require design refinement. This dataset construction is closely aligned with the goal of PartSense-IP. Instead of evaluating prototype quality using only global image similarity, IP-ProtoSense provides multi-view sensor observations, depth information, part-level labels, and local inconsistency masks. Therefore, it supports a more comprehensive evaluation of sensor-based IP prototype assessment, including consistency scoring, inconsistency detection, localization, and interpretable feedback generation.

**Figure 3:**
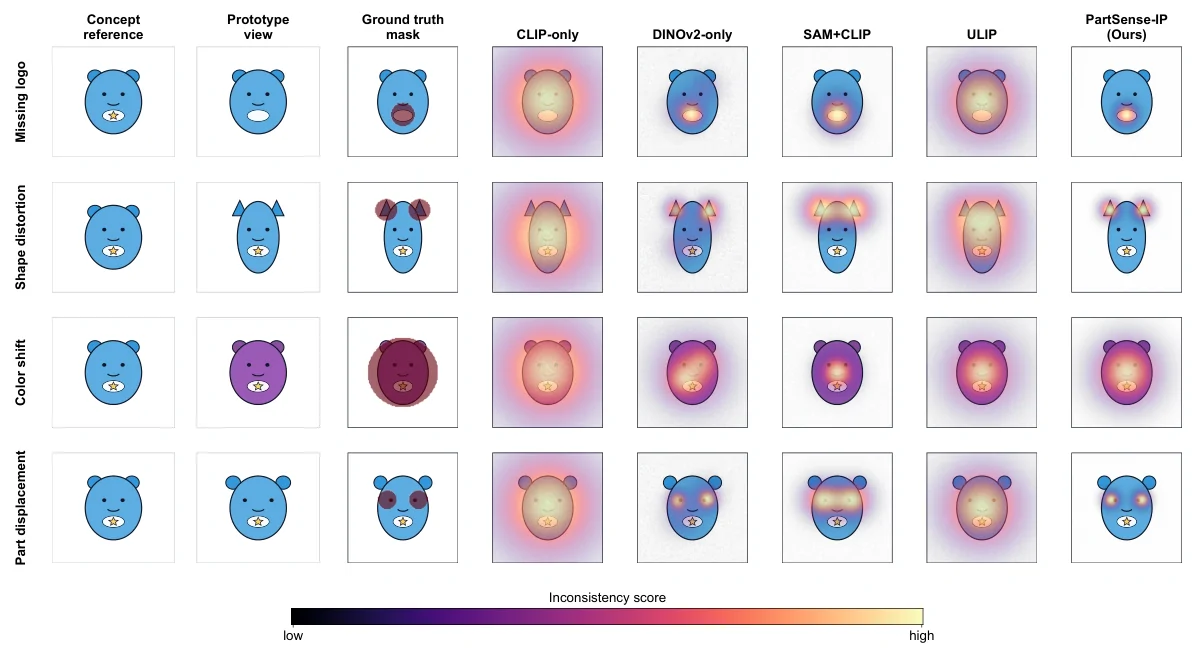
Qualitative comparison of inconsistency localization results under representative design-deviation cases. Four types of deviations are shown, including missing logo, shape distortion, color shift, and part displacement.

Figure 3 presents representative qualitative examples for evaluating the inconsistency localization capability of different methods. Four typical design-deviation cases are considered, including missing logo, shape distortion, color shift, and part displacement. These cases correspond to common prototype-review problems in IP product design, where the prototype may preserve the overall object category but lose or distort identity-critical visual elements. For each case, the clean concept reference, the deviated prototype view, the ground-truth inconsistency mask, and the localization results of different methods are demonstrated.

Specifically, the CLIP-only baseline produces relatively global and diffuse activation maps. This is because CLIP mainly captures image-level visual–semantic similarity and is not specifically designed for local inconsistency localization. As a result, it can indicate that the prototype is visually different from the concept reference, but it cannot precisely determine whether the deviation comes from a missing logo, distorted ears, shifted facial components, or a color change. This limitation is particularly evident in the missing-logo and part-displacement cases, where the inconsistent regions are small but identity-critical. The DINOv2-only scheme provides stronger local visual sensitivity than the CLIP-only one. It can respond to local appearance differences and produces more concentrated responses in some cases. However, its activation maps may include irrelevant or noisy regions because it does not explicitly incorporate design semantics, part-level correspondence, or view-level stability. For example, in the shape-distortion and part-displacement cases, DINOv2 can highlight visually changed regions, but the response is less stable and may spread to nearby non-defective areas. The SAM + CLIP method improves localization by introducing part-level segmentation before semantic matching. Compared with CLIP-only, it can better associate the inconsistency with object components such as the logo, ears, or facial parts. Nevertheless, its localization is still relatively coarse because it operates mainly on 2D segmented regions and does not use RGB-D sensing, multi-view fusion, or cross-view stability constraints. Therefore, when the inconsistent part is small or when the deviation affects both geometry and appearance, the detected region may be over-expanded. The ULIP baseline introduces 3D multimodal representation and is able to capture object-level correspondence between visual, textual, and 3D information. However, it tends to focus on global object-level consistency rather than fine-grained IP-specific design elements. Therefore, it may generate broad responses over the whole prototype and is less sensitive to small local deviations, such as missing symbolic logos or displaced facial features.

In contrast, our proposed PartSense-IP framework generates more accurate and localized inconsistency maps. For the missing-logo case, the highlighted region is concentrated around the absent symbolic element. For the shape-distortion case, the response is mainly located on the deformed ears. For the color-shift case, the heatmap covers the main body region where the color deviation occurs. For the part-displacement case, the highlighted regions correspond to the shifted facial components. These results show that PartSense-IP can better distinguish different sources of prototype inconsistency by jointly using part-aware visual representation, vision–language semantic alignment, RGB-D sensing, and multi-view consistency fusion.

**Figure 4:**
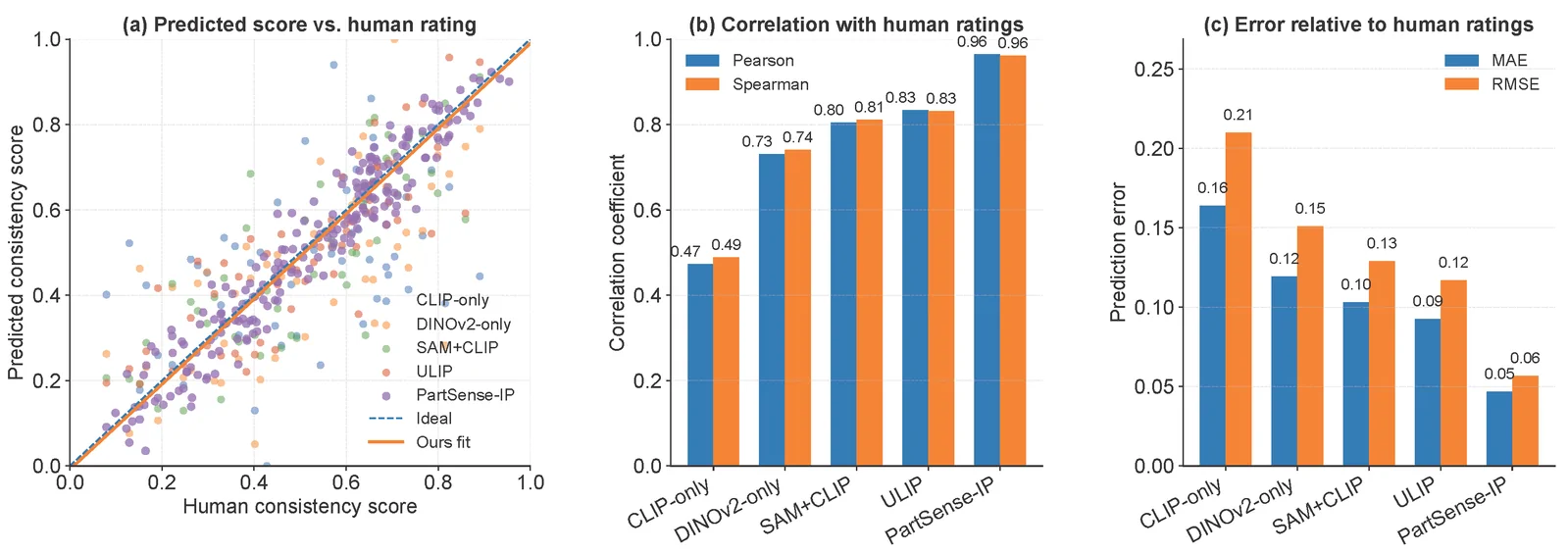
Overall consistency scoring performance. (**a**) Predicted consistency scores versus human-annotated consistency ratings. (**b**) Pearson and Spearman correlation coefficients between predicted scores and human ratings. (**c**) Prediction errors measured by MAE and RMSE.

Figure 4 evaluates the overall consistency scoring performance of different methods by comparing the predicted consistency scores with human-annotated ratings. The objective of this experiment is to verify whether each method can provide a reliable scalar score that reflects the degree of visual–semantic consistency between the reference concept design and the sensor-captured prototype. As shown in Figure 4a, the predicted scores of the proposed PartSense-IP framework are more tightly distributed around the ideal diagonal line than those of the baseline methods. This indicates that the proposed score $S_{\mathrm{IP}}$ is more consistent with human perception. In contrast, CLIP-only exhibits a much more scattered distribution. Although CLIP can capture global visual–semantic similarity, it is less reliable for fine-grained IP prototype evaluation because small but identity-critical deviations, such as missing logos or local part displacement, may not significantly change the global semantic embedding. DINOv2-only improves the alignment with human ratings by extracting stronger visual features, but it still lacks explicit semantic reasoning and part-level design interpretation. SAM + CLIP and ULIP further improve the score prediction by introducing part-level matching and 3D multimodal representation, respectively. However, their predictions remain less stable than PartSense-IP because they do not jointly model part-aware visual features, semantic design alignment, RGB-D sensing, and multi-view consistency.

Figure 4b compares the Pearson and Spearman correlation coefficients between predicted scores and human ratings. CLIP-only obtains the lowest correlation, with Pearson and Spearman coefficients of 0.47 and 0.49, respectively. DINOv2-only improves the correlation to 0.73 and 0.74, showing that dense visual features are more effective than global image-level semantics for visual consistency evaluation. SAM + CLIP further increases the correlation to 0.80 and 0.81 by introducing part-level segmentation and matching. ULIP achieves Pearson and Spearman correlations of 0.83 and 0.83, indicating the benefit of incorporating 3D multimodal representation. The proposed PartSense-IP achieves the highest Pearson and Spearman correlations, reaching 0.96 and 0.96, respectively. This demonstrates that the proposed fusion strategy provides a score that is much more aligned with human design judgments. Figure 4c reports the prediction errors measured by MAE and RMSE. CLIP-only produces the largest error, with an MAE of 0.16 and an RMSE of 0.21. DINOv2-only reduces the error to 0.12 and 0.15, while SAM + CLIP further reduces it to 0.10 and 0.13. ULIP achieves an MAE of 0.09 and an RMSE of 0.12. In comparison, PartSense-IP obtains the lowest prediction error, with an MAE of 0.05 and an RMSE of 0.06. These results confirm that the proposed method not only improves correlation with human ratings but also reduces absolute scoring deviation.

**Figure 5:**
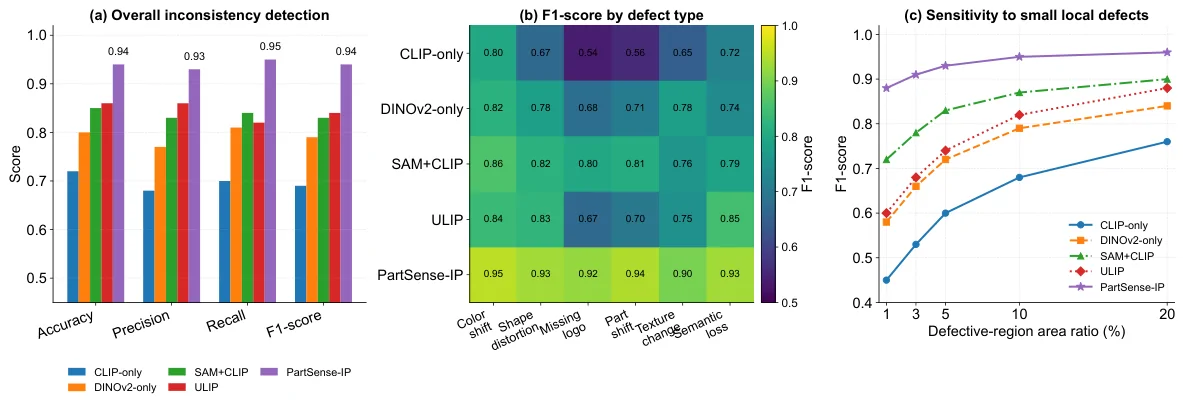
Inconsistency. (**a**) Overall detection results measured by accuracy, precision, recall, and F1-score. (**b**) F1-score comparison under different defect types, including color shift, shape distortion, missing logo, part shift, texture change, and semantic loss. (**c**) F1-score under different defective-region area ratios.

Figure 5 evaluates the inconsistency detection performance of different methods. Unlike Figure 4, which focuses on predicting a continuous consistency score, this experiment further examines whether each method can correctly identify the existence and category of design inconsistencies in IP prototypes. Figure 5a reports the overall binary detection performance in terms of accuracy, precision, recall, and F1-score. CLIP-only obtains the lowest performance, with an accuracy of 0.72 and an F1-score of 0.69. This is mainly because CLIP relies on global visual–semantic similarity and is not specifically designed to detect fine-grained prototype deviations. DINOv2-only improves the F1-score to 0.79, indicating that dense visual features are more effective for capturing local appearance changes. SAM + CLIP further increases the F1-score to 0.83 by introducing part-level segmentation and matching. ULIP achieves a comparable F1-score of 0.84, benefiting from 3D multimodal representation. In comparison, the proposed PartSense-IP achieves the best performance, with an accuracy of 0.94, precision of 0.93, recall of 0.95, and F1-score of 0.94. This confirms that the proposed part-aware visual–semantic fusion strategy is more reliable for identifying inconsistent prototypes.

Figure 5b provides a more detailed comparison across different defect types. The results show that CLIP-only performs reasonably on global color shift but is much weaker on small or local defects, such as missing logo and part shift, where its F1-scores drop to 0.54 and 0.56, respectively. DINOv2-only improves the detection of local visual changes, but it still lacks explicit semantic interpretation of identity-critical design elements. SAM + CLIP performs better on missing logo and part shift because the segmentation step provides part-level localization. However, it remains less effective for texture change and semantic loss, as it does not jointly consider multi-view stability and RGB-D sensing. ULIP performs well on semantic loss due to its multimodal 3D representation, but it is less sensitive to small local elements such as missing logos. In contrast, PartSense-IP consistently achieves the highest F1-score across all defect types, including color shift, shape distortion, missing logo, part shift, texture change, and semantic loss. In particular, the strong performance on missing logo and part shift demonstrates the advantage of explicitly modeling part-level IP identity cues.

Figure 5c further evaluates the sensitivity of different methods to the size of defective regions. This experiment is important because many IP-related inconsistencies occur in small but semantically important regions, such as logos, facial details, accessories, or decorative patterns. As the defective-region area ratio decreases, all baseline methods suffer from performance degradation. CLIP-only is the most sensitive to small defect regions, achieving an F1-score of only 0.45 when the defect occupies 1% of the object area. DINOv2-only and ULIP perform better but still show clear degradation. SAM + CLIP is more robust due to its part-level segmentation, but its F1-score remains lower than that of PartSense-IP. The proposed method maintains an F1-score of 0.88 even when the defective region occupies only 1% of the object area, and its performance increases to 0.96 when the region ratio reaches 20%. This shows that PartSense-IP is particularly effective for detecting small local defects that are often critical for IP visual identity preservation.

**Figure 6:**
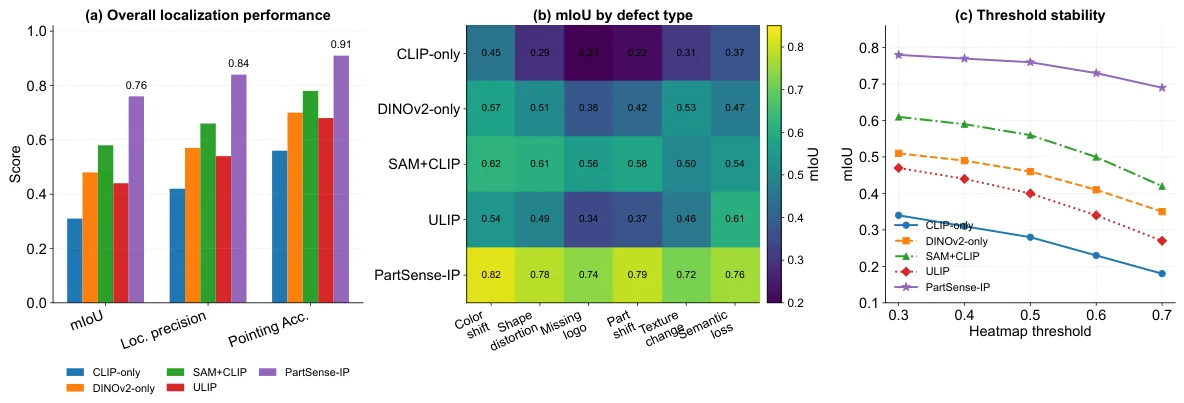
Localization performance of inconsistency maps. (**a**) Overall localization results measured by mIoU, localization precision, and pointing accuracy. (**b**) mIoU comparison under different defect types, including color shift, shape distortion, missing logo, part shift, texture change, and semantic loss. (**c**) Localization stability under different heatmap binarization thresholds.

Figure 6 evaluates the localization performance of different methods for inconsistency maps. Different from the detection experiment in Figure 5, this experiment focuses on whether the predicted inconsistency regions are spatially aligned with the ground-truth deviation masks. Three metrics are used, including mean Intersection-over-Union (mIoU), localization precision, and pointing accuracy. The mIoU measures the overlap between the predicted inconsistency map and the ground-truth mask, localization precision evaluates whether the highly activated regions fall inside the true inconsistent area, and pointing accuracy measures whether the maximum-response point is located within the ground-truth region. As shown in Figure 6a, CLIP-only obtains the weakest localization performance, with an mIoU of 0.31, localization precision of 0.42, and pointing accuracy of 0.56. This is expected because CLIP mainly provides global visual–semantic similarity and is not designed to generate spatially accurate local inconsistency maps. DINOv2-only improves the localization performance, reaching an mIoU of 0.48 and a pointing accuracy of 0.70. This indicates that dense visual features are more useful for detecting local visual changes. However, DINOv2 still lacks explicit part-level semantic correspondence and may activate visually similar but semantically irrelevant regions. SAM + CLIP further improves the mIoU to 0.58 and the pointing accuracy to 0.78, showing the benefit of part-level segmentation. ULIP achieves moderate localization performance, with an mIoU of 0.44 and a pointing accuracy of 0.68, because it mainly captures object-level 3D multimodal similarity and is less sensitive to fine-grained local deviations. In comparison, the proposed PartSense-IP achieves the best localization results, with an mIoU of 0.76, localization precision of 0.84, and pointing accuracy of 0.91.

Figure 6b further compares the mIoU values under different defect types. CLIP-only performs poorly on local and identity-critical defects, such as missing logo and part shift, where its mIoU values are only 0.20 and 0.22, respectively. DINOv2-only improves the localization of visual changes but still suffers from limited semantic understanding, especially for missing logo and semantic loss. SAM + CLIP provides better results for missing logo and part shift because the segmentation step introduces part-level structure. However, its performance remains limited for texture change and semantic loss, where purely 2D part matching is insufficient. ULIP performs relatively better on semantic loss, but its localization of small local elements remains weak. In contrast, PartSense-IP consistently achieves the highest mIoU across all defect types, including color shift, shape distortion, missing logo, part shift, texture change, and semantic loss. In particular, the proposed method obtains mIoU values of 0.74 and 0.79 for missing logo and part shift, respectively, demonstrating its advantage in locating small but important IP identity cues.

Figure 6c evaluates the stability of localization results under different heatmap binarization thresholds. A reliable localization method should not be overly sensitive to a specific threshold. The results show that the mIoU of CLIP-only, DINOv2-only, SAM + CLIP, and ULIP decreases rapidly as the threshold increases, indicating that their inconsistency maps are either too diffuse or unstable. In contrast, PartSense-IP maintains consistently high mIoU values across the tested threshold range. Even when the threshold increases to 0.70, PartSense-IP still achieves an mIoU of 0.69, which is higher than the best results of most baselines at lower thresholds. This confirms that the proposed inconsistency maps are sharper, more stable, and better aligned with the true defect regions. Overall, the improvement comes from the joint modeling of part-aware visual representation, vision–language semantic alignment, RGB-D sensing, and multi-view consistency fusion. These results are important for practical IP product prototype evaluation because designers require not only a consistency score but also spatially interpretable feedback indicating which regions should be refined.

### 4.4. Statistical Significance Analysis

To complement the deterministic point-estimate comparison, we further examine the statistical reliability of the improvements obtained by PartSense-IP. Grounding DINO + CLIP is selected as the strongest competing baseline because it achieves the best overall performance among the evaluated non-proposed methods. Since the pretrained backbones are frozen and the PVCF procedure uses fixed parameters, the principal test results are deterministic for a given input set. We therefore report one point estimate for each method rather than run-to-run means and standard deviations. Statistical uncertainty is instead assessed through asset-level bootstrap resampling with 1000 repetitions. A two-sided Wilcoxon signed-rank test is additionally applied to the paired per-sample results.

As shown in Table 5, all bootstrap confidence intervals of the paired improvement remain above zero. The two-sided Wilcoxon signed-rank tests also yield p<0.001 for all four principal metrics. Since the principal evaluation pipeline is deterministic, these statistics do not measure run-to-run variation or sensitivity to random initialization. Instead, they quantify the uncertainty associated with the finite test set and test whether the paired per-sample improvements are consistently greater than zero. The results therefore provide statistical support for the observed improvements in consistency scoring, inconsistency detection, and localization.

### 4.5. Synthetic-to-Real Generalization

Although controlled prototype variants provide accurately traceable inconsistency regions and enable reproducible evaluation, isolated synthetic deviations may be easier to identify than inconsistencies observed in practical prototype review. Moreover, because the controlled variants are generated according to predefined deviation categories, it is necessary to verify that the reported performance does not result from a favorable correspondence between these editing operations and the shape, color, visual, and semantic measurements adopted by PartSense-IP. We therefore conduct an additional synthetic-to-real generalization evaluation using the independently captured real-world challenge subset.

The controlled synthetic test subset contains prototype variants generated through isolated and traceable modifications, including color shift, shape distortion, missing symbolic elements, part displacement, texture inconsistency, and semantic identity loss. In contrast, the real-captured challenge subset contains physical or 3D-printed prototypes whose differences from the corresponding concept references are not restricted to a single predefined operator. The real samples may exhibit coupled variations in geometry, component placement, color, material appearance, surface texture, decorative completeness, illumination, and sensing conditions. Their inconsistent regions also tend to have less sharply defined boundaries than those in the controlled subset.

It is also important to distinguish genuine prototype inconsistency from the normal domain difference between stylized concept artwork and a physical object. The concept image may contain simplified shading, non-photorealistic textures, or exaggerated contours, whereas the captured prototype includes perspective effects, real materials, illumination-dependent reflections, and sensor noise. The evaluation is therefore based on human judgments of identity-critical consistency rather than pixel-level appearance equivalence. Such normal domain differences are not treated as inconsistencies unless they change the intended shape, color identity, symbolic component, local design feature, or semantic character of the prototype. Additionally, real manufacturing deviations often vary continuously over a surface. Unlike the exact binary masks obtained from controlled synthetic editing operations, the real-captured masks represent the principal perceptually affected regions identified by the annotators and may contain uncertainty near gradual boundaries. Consequently, localization metrics on the real subset should be interpreted as in approximate agreement with human-identified affected regions rather than as exact recovery of a physically binary modification boundary.

To ensure a strict evaluation protocol, the real-captured samples are not used for training, validation, PVCF coefficient selection, decision threshold determination, or preprocessing-parameter adjustment. All methods retain the same configurations determined using only the synthetic training and validation subsets. The resulting models are directly evaluated on the real-captured challenge subset without retraining or parameter reselection. Therefore, the comparison evaluates whether the methods generalize beyond the controlled deviation operators used during benchmark construction.

As shown in Table 6, all evaluated methods exhibit lower performance on the real-captured challenge subset than on the controlled synthetic test subset. This performance reduction is expected because practical prototype differences are generally less isolated and are affected by coupled geometric and appearance variations, illumination changes, material reflections, occlusion, and sensing noise. In addition, the boundaries of real inconsistencies are often more ambiguous, which particularly affects localization-oriented metrics such as mIoU and pointing accuracy. Nevertheless, PartSense-IP consistently achieves the best performance on both subsets. On the real-captured challenge subset, it obtains a Pearson correlation of 0.872, an F1-score of 0.836, an mIoU of 0.594, and a pointing accuracy of 0.774. Compared with the strongest competing SAM + CLIP baseline, PartSense-IP improves Pearson correlation by 0.066, F1-score by 0.057, mIoU by 0.086, and pointing accuracy by 0.073. The relatively pronounced improvements in the localization metrics indicate that part-aware correspondence and cross-view consistency remain useful when deviations are no longer generated by isolated synthetic operators.

Since no model configuration, PVCF coefficient, or decision threshold is adjusted using the real-captured samples, these results provide evidence that the proposed framework does not merely exploit the controlled deviation-generation procedure. Instead, the visual, semantic, and multi-view cues learned or configured on the synthetic subsets retain their effectiveness under independently captured prototype observations. The remaining synthetic-to-real performance gap also reflects the fundamental domain difference between stylized 2D concept artwork and sensor-captured physical prototypes, including perspective, material, illumination, self-occlusion, and sensing effects. This result indicates that the 2D-to-3D domain gap remains challenging and that the controlled benchmark cannot fully reproduce the complexity of practical design review. The current real-captured subset should therefore be regarded as an initial challenge evaluation rather than a complete industrial-scale benchmark. Future work will extend the dataset with larger collections of factory-produced prototypes, more diverse fabrication processes, naturally occurring compound deviations, and annotations from professional product designers.

### 4.6. Feedback-Quality Evaluation

The localization metrics evaluate where an inconsistency occurs, but they do not directly determine whether the generated feedback identifies the correct design part and inconsistency attribute. We therefore conducted a separate quantitative evaluation of the structured feedback output using the part-level and inconsistency-category annotations already included in IP-ProtoSense. No additional labels or test-set tuning were introduced for this evaluation.

For each generated report, the structured output contains an affected part label and a dominant inconsistency-type label. Affected-part accuracy measures the proportion of samples for which the predicted part agrees with the annotated identity-critical part. Inconsistency-type accuracy measures whether the report correctly identifies the dominant deviation as shape, color, local visual/texture, or semantic inconsistency. Joint part–type accuracy requires both the affected part and the inconsistency type to be correctly identified. When more than one annotated deviation is present in a real-captured sample, a prediction is treated as correct if it matches one of the annotated primary part–deviation pairs.

As reported in Table 7, the feedback generator correctly identifies the affected design part in 91.8% of the controlled test samples and the dominant inconsistency type in 90.4% of the samples. The joint part–type accuracy is 86.1%, indicating that most reports simultaneously describe both where the inconsistency occurs and which design attribute is affected. The results are lower on the real-captured challenge subset, where multiple deviations may occur simultaneously and the distinction among shape, texture, material appearance, and semantic inconsistency is less clear. Nevertheless, the generated reports achieve an affected-part accuracy of 84.2%, an inconsistency-type accuracy of 81.7%, and a joint accuracy of 75.8%. These results directly complement the localization evaluation and show that the feedback module generally converts the underlying component scores into correct and design-relevant diagnostic statements. A larger user study with professional industrial designers would still be valuable for evaluating long-term usefulness in practical design workflows.

### 4.7. Ablation Study

**Figure 7:**
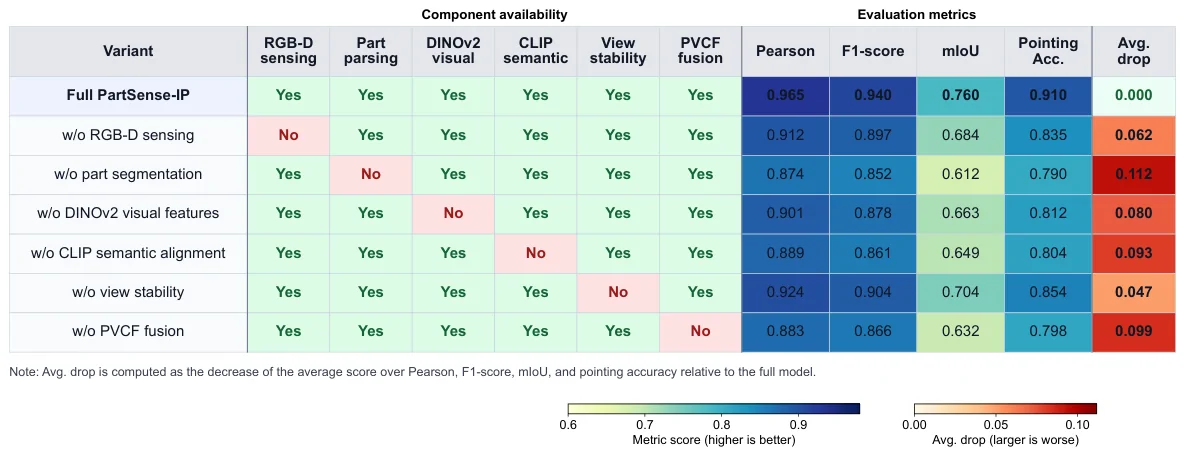
Ablation study of the proposed PartSense-IP framework. The full model achieves the best performance across Pearson correlation, F1-score, mIoU, and pointing accuracy. Removing part segmentation, CLIP-based semantic alignment, or PVCF fusion causes clear performance degradation, demonstrating the importance of part-aware representation, vision–language semantic reasoning, and structured consistency fusion. RGB-D sensing and view stability further improve localization accuracy and robustness.

Figure 7 presents the ablation study of the proposed PartSense-IP framework. The purpose of this experiment is to verify the contribution of each key module, including RGB-D sensing, part parsing, DINOv2-based visual feature extraction, CLIP-based semantic alignment, cross-view stability modeling, and the proposed PVCF fusion strategy. The full PartSense-IP framework achieves the best performance across all metrics, with a Pearson correlation of 0.965, an F1-score of 0.940, an mIoU of 0.760, and a pointing accuracy of 0.910.

Removing RGB-D sensing leads to a clear performance drop. The Pearson correlation decreases from 0.965 to 0.912, while the mIoU decreases from 0.760 to 0.684. This indicates that depth and multi-view sensing information are important for stabilizing prototype evaluation, especially when the inconsistency involves geometric deformation or view-dependent appearance changes. Without RGB-D sensing, the system relies more heavily on 2D visual observations and becomes less effective in spatially locating inconsistent regions.

The largest degradation occurs when part segmentation is removed. In this case, the F1-score decreases to 0.852, the mIoU decreases to 0.612, and the average performance drop reaches 0.112. This confirms that part-aware representation is one of the most important components of PartSense-IP. Since IP product consistency often depends on identity-critical local elements, such as logos, facial features, ears, accessories, and decorative regions, global image-level comparison is insufficient. Part parsing allows the framework to evaluate whether each design-relevant component is preserved in the prototype.

Removing DINOv2-based visual features also reduces performance, with the mIoU decreasing from 0.760 to 0.663 and the pointing accuracy decreasing from 0.910 to 0.812. This demonstrates that dense visual representation is useful for capturing local appearance, texture, and shape differences. Without this module, the framework loses part of its ability to distinguish subtle visual deviations within segmented regions.

When CLIP-based semantic alignment is removed, the Pearson correlation decreases to 0.889 and the F1-score decreases to 0.861. This result shows that visual similarity alone cannot fully represent IP design consistency. Some prototype deviations are semantic rather than purely visual, such as style mismatch, symbolic identity loss, or inconsistency with the textual design description. The semantic alignment module helps PartSense-IP connect sensor-captured observations with the intended design meaning.

Removing view stability modeling causes a smaller but still noticeable degradation. The average performance drop is 0.047, which suggests that cross-view consistency mainly improves robustness rather than replacing the role of visual or semantic features. This module is particularly useful when a local design element is visible only from specific viewpoints or when a prototype shows inconsistent appearance across views.

Finally, removing the PVCF fusion module leads to an average performance drop of 0.099. This indicates that simply combining the extracted features without structured fusion is insufficient. The PVCF module plays an important role in integrating shape, color, part-level visual similarity, semantic alignment, and view stability into a unified consistency score and localization output.

Consequently, the ablation results demonstrate that each module contributes to the final performance of PartSense-IP. Among them, part segmentation, semantic alignment, and PVCF fusion have particularly strong effects, while RGB-D sensing and view stability further improve robustness and localization accuracy. These results validate the main design motivation of the proposed framework: reliable IP prototype evaluation requires a joint modeling of sensor information, part-aware visual representation, vision–language semantics, and multi-view consistency.

The preceding module-removal experiments evaluate the contribution of individual sensing and representation components. However, since PartSense-IP employs pretrained segmentation, dense visual, and vision–language models, these experiments alone do not fully distinguish the contribution of PVCF from the benefit of combining SAM, DINOv2, and CLIP representations. We therefore conduct a backbone-controlled fusion comparison. In this comparison, all variants use exactly the same SAM-generated part masks, DINOv2 visual features, CLIP semantic embeddings, RGB-D observations, and test samples. No pretrained encoder or input modality is changed. The compared variants differ only in how the resulting consistency measurements are organized and aggregated. Therefore, any performance difference in this experiment can be attributed to the fusion strategy rather than to the underlying pretrained models. To address whether the baseline performance is limited by their original global or object-level configurations, we further conduct a controlled part-aware backbone comparison. Part-CLIP, Part-DINOv2-MNN, and Part-ULIP use exactly the same SAM-generated concept and prototype masks, part-visibility rules, null assignments, multi-view observations, and uniform part/view aggregation. Therefore, the three variants differ only in the representation and local matching backbone.

As shown in Table 8, applying the same part-decomposition pipeline substantially strengthens all three representation backbones. Among the controlled variants, Part-DINOv2-MNN achieves the best performance, with a Pearson correlation of 0.940, an F1-score of 0.915, an mIoU of 0.713, and a pointing accuracy of 0.869. This confirms that dense spatial correspondence is important for detecting local prototype deviations and that the weaker performance of the original DINOv2 baseline was partly attributable to its global pooling configuration. Part-CLIP also benefits from replacing global image-level similarity with local patch-token comparison, while Part-ULIP improves after the fused point cloud is decomposed into part-level subsets. Nevertheless, all three controlled variants remain below the complete PartSense-IP framework. In particular, PartSense-IP improves the mIoU from 0.713 to 0.760 and the pointing accuracy from 0.869 to 0.910 relative to the strongest controlled backbone. These improvements indicate that a strong local backbone alone is insufficient and that complementary shape, color, visual, semantic, identity-aware, and cross-view evidence remains beneficial.

As shown in Table 9, simply averaging the outputs of the pretrained components produces the lowest performance, although this variant uses the same SAM, DINOv2, CLIP, and RGB-D information as the complete framework. Introducing concept-to-prototype part correspondence improves the Pearson correlation from 0.892 to 0.931 and the mIoU from 0.646 to 0.702, demonstrating that local identity-critical regions cannot be adequately represented by global score aggregation. Learning a linear combination of the same component scores further improves the results. Nevertheless, learned linear fusion remains inferior to the complete PVCF framework, particularly for localization metrics. Compared with learned linear fusion, the full PVCF improves the mIoU from 0.728 to 0.760 and the pointing accuracy from 0.879 to 0.910. Since the underlying pretrained models and sensor observations remain unchanged, these improvements isolate the contribution of the proposed hierarchical fusion mechanism. The results demonstrate that the performance gain cannot be explained solely by combining DINOv2, CLIP, and SAM. Instead, explicit concept-to-prototype part correspondence, identity-aware part weighting, and cross-view stability modeling are required to convert the generic pretrained representations into reliable prototype-level scoring and localized inconsistency assessment.

To further assess whether the fixed weighted formulation of PVCF is competitive with more flexible learning-based aggregation mechanisms, we additionally compare it with MLP, attention-based, Transformer-based, and graph-based fusion strategies. This comparison is conducted under a strictly controlled setting. All variants use the same SAM-generated part masks, DINOv2 visual features, CLIP semantic embeddings, RGB-D observations, part correspondences, and dataset splits. Only the module used to aggregate the resulting component, part, and view-level consistency variables is replaced. For MLP fusion, the four consistency components of each matched part are processed by a two-hidden-layer perceptron. Attention-based fusion learns sample-dependent weights over shape, color, dense-visual, and semantic consistency. Transformer fusion treats the part-view consistency representations as a sequence of tokens and applies a two-layer Transformer encoder. Graph-based aggregation represents each part–view pair as a node, with edges connecting the same part across views and related parts within each view. All learnable variants are trained only on the training split, with model selection and early stopping performed on the validation split. No test-set sample is used for architecture selection or parameter optimization.

As shown in Table 10, nonlinear and structure-aware fusion mechanisms generally improve over learned linear fusion, confirming that interactions among shape, color, visual, semantic, part, and view-level evidence can be beneficial. MLP fusion achieves a Pearson correlation of 0.957 and an mIoU of 0.746, while attention-based fusion further improves these values to 0.962 and 0.755, respectively. The Transformer and graph-based variants provide the strongest results among the learnable alternatives. Transformer fusion achieves the highest Pearson correlation of 0.968, whereas graph-based part aggregation obtains the highest F1-score, mIoU, and pointing accuracy of 0.943, 0.763, and 0.912, respectively. Nevertheless, their improvements over the fixed PVCF formulation are marginal. Relative to the strongest graph-based variant, PVCF differs by only 0.003 in F1-score, 0.003 in mIoU, and 0.002 in pointing accuracy. Importantly, PVCF introduces no trainable fusion parameters and retains an explicit correspondence between the final decision and its shape, color, local-visual, semantic, part-importance, and cross-view components. In contrast, the learned nonlinear models require additional task-specific optimization and generally provide less direct attribute-level diagnosis. Therefore, the results do not establish that fixed PVCF is universally optimal among all possible fusion functions. Instead, they show that PVCF provides a favorable trade-off among accuracy, model complexity, data dependence, and interpretability, while remaining competitive with substantially more complex learned fusion architectures.

### 4.8. Sensitivity to PVCF Fusion Weights

To examine whether the performance of PartSense-IP depends on a specific manually assigned coefficient combination, we compare the default PVCF setting with uniform fusion and four deliberately biased configurations that emphasize shape, color, dense visual similarity, or semantic consistency. We further implement a validation-learned linear fusion variant. For this variant, the four component scores are retained as input variables, and the coefficients are optimized on the validation split under the constraints $\alpha_{i}\geq 0$ and $\sum_{i=1}^{4}\alpha_{i}=1$. No test sample is used for coefficient selection or optimization.

As reported in Table 11, uniform fusion already achieves competitive performance, indicating that the effectiveness of PartSense-IP does not depend on a narrowly tuned coefficient combination. In contrast, excessively emphasizing a single component generally reduces performance because different prototype inconsistencies require complementary shape, color, local visual, and semantic evidence. The validation-learned coefficients are (0.24,0.18,0.32,0.26), which are close to the adopted fixed coefficients (0.25,0.20,0.30,0.25). The learned variant provides only marginal improvements. Therefore, the fixed PVCF setting is retained as the default because it provides near-optimal performance while remaining transparent and avoiding additional dataset-dependent optimization.

### 4.9. Robustness Evaluation

To strengthen the robustness evaluation against more competitive multimodal alternatives, we extend the original baseline set with BLIP-2 [21], Grounding DINO + CLIP [12,24,25,37], and OpenShape [41]. These methods represent three complementary categories that were not sufficiently covered by the original comparison: stronger global vision–language alignment, language-guided part grounding, and open-world 3D vision–language representation, respectively. All methods are evaluated under identical input perturbations and test samples, and no method is reconfigured separately for an individual perturbation condition.

All perturbations are applied only to the test data after model configuration and parameter selection, and identical perturbed samples are used for all compared methods. Reduced-view settings retain either the front, left, and right views or only the front view. Low-light and shadow conditions are generated by RGB intensity attenuation with mild sensor noise and soft spatial masks, respectively. Depth degradation is simulated using Gaussian depth noise, random invalid pixels, and spatially contiguous depth removal. Occlusion is introduced by masking approximately 10%, 20%, or 40% of the visible object region, while background clutter is produced using indoor scenes with additional distractor objects. Pose errors are applied to the camera extrinsic parameters using rotational perturbations of 5°, 10°, or 20° together with translation jitter within ±20 mm. Text perturbations include removing an identity-critical attribute, inserting an irrelevant but plausible attribute, replacing a specific description with a generic one, or removing the text input entirely. Three random realizations are evaluated for each stochastic condition, and the reported results are averaged over these realizations.

As shown in Table 12, the stronger additional baselines improve over their corresponding original counterparts. BLIP-2 generally outperforms CLIP-only under appearance-related perturbations, while Grounding DINO + CLIP provides greater robustness than SAM + CLIP because language-guided grounding improves the identification of identity-critical regions. OpenShape also improves over ULIP under view reduction, depth corruption, and pose errors, confirming the benefit of a stronger 3D multimodal representation. Nevertheless, PartSense-IP achieves the highest F1-score under every tested sensing condition. Its average perturbed-setting F1-score is 0.89, compared with 0.81 for both Grounding DINO + CLIP and OpenShape. It also exhibits the smallest decrease from the clean setting, with a drop of 0.05. These results indicate that the robustness advantage is preserved when the comparison is extended beyond the original CLIP, DINOv2, SAM + CLIP, and ULIP baselines.

Table 13 further shows that the performance advantage remains under practical occlusion, clutter, pose-calibration, and text-description perturbations. Grounding DINO + CLIP is the strongest added baseline, achieving an average F1-score of 0.78, followed by OpenShape with 0.75. PartSense-IP achieves an average F1-score of 0.85 and remains the best-performing method for every perturbation condition. The advantage becomes particularly evident under severe perturbations. For example, under 40% occlusion, PartSense-IP achieves an F1-score of 0.79, compared with 0.68 for Grounding DINO + CLIP and 0.63 for OpenShape. Under a 20° rotation error, the corresponding scores are 0.80, 0.73, and 0.69, respectively. Even without a textual design description, PartSense-IP retains an F1-score of 0.81, indicating that its performance does not depend exclusively on the vision–language semantic branch.

### 4.10. 3D-to-2D Inconsistency Map Projection

**Figure 8:**
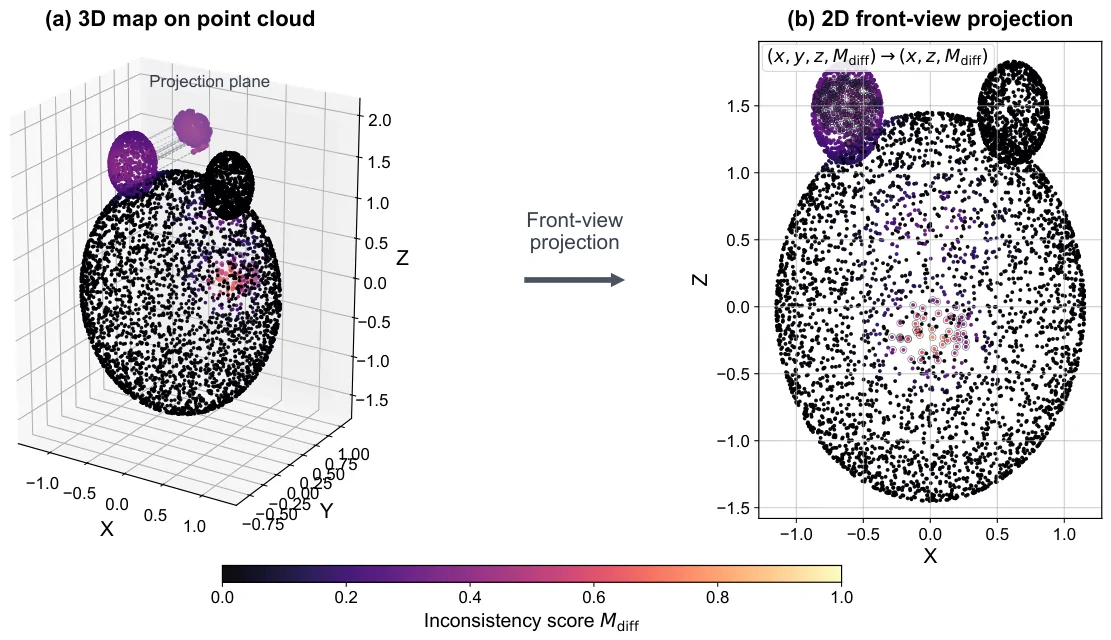
Visualization of the 3D-to-2D inconsistency map projection. (**a**) The predicted inconsistency map $M_{\mathrm{diff}}$ is projected onto the sensed 3D prototype point cloud, where each point is colored according to its inconsistency score. The projection plane and projection rays illustrate how the 3D inconsistency representation is mapped to a front-view plane. (**b**) The corresponding 2D front-view projection, where $\left(x,y,z,M_{\mathrm{diff}}\right)$ is transformed into $\left(x,z,M_{\mathrm{diff}}\right)$. The projected map provides an interpretable view-level explanation of the detected local inconsistencies and supports designer-oriented prototype refinement.

In addition to numerical evaluation, we further visualize the spatial inconsistency map generated by PartSense-IP to demonstrate its interpretability for prototype inspection. The objective of this experiment is to show how the predicted inconsistency score can be mapped back to the sensed prototype geometry and further projected onto a 2D view for human-readable design feedback. This is important because practical IP product evaluation requires not only a final consistency score, but also an explicit indication of where the prototype deviates from the intended design. Figure 8 illustrates the 3D-to-2D projection of the inconsistency map $M_{\mathrm{diff}}$. In Figure 8a, each point in the prototype point cloud is assigned an inconsistency score. Formally, the spatial inconsistency representation can be written as $\left(x,y,z,M_{\mathrm{diff}}\right)$, where ((x,y,z)) denotes the 3D coordinate of a sensed surface point and $M_{\mathrm{diff}}$ denotes the predicted inconsistency intensity at that location. Higher scores indicate regions that are more likely to be inconsistent with the reference IP design. The visualization shows that the high-response regions are concentrated around identity-critical local components, such as the ear region and the front symbolic region, rather than being distributed uniformly over the entire object surface.

To provide a more intuitive view-level explanation, the 3D inconsistency map is further projected onto a 2D front-view plane, as shown in Figure 8b. The projection can be interpreted as $\left(x,y,z,M_{\mathrm{diff}}\right)\to \left(x,z,M_{\mathrm{diff}}\right)$, where the depth coordinate along the viewing direction is removed while the inconsistency score is preserved. The projection rays and the front-view projection plane in Figure 8a illustrate this mapping process. The resulting 2D map provides a compact and human-readable representation of the detected inconsistencies, making it easier for designers to identify which local regions should be refined. The visual results confirm that PartSense-IP can transform abstract consistency scores into spatially interpretable feedback. Compared with global similarity scores, the projected inconsistency map offers more actionable information because it localizes the problematic parts on the prototype surface. This capability is particularly useful for IP product design, where small local components, such as logos, facial features, ears, accessories, and decorative patterns, may carry important semantic identity. Therefore, the 3D-to-2D inconsistency visualization provides an interpretable bridge between sensor-level prototype observation and designer-oriented revision guidance.

### 4.11. Discussion

Although PartSense-IP is evaluated using the IP-ProtoSense protocol, the framework is not tied to a predefined product category or a fixed set of prototype-deviation operators. Its inputs are generic concept images, optional textual descriptions, and multi-view RGB-D observations, while the consistency variables are defined in terms of shape, color, local visual appearance, semantic alignment, and cross-view stability. These quantities are not category-specific and can, in principle, be applied to previously unseen objects and design styles, provided that identity-critical parts can be identified and corresponding sensor views are available. The controlled deviation categories in IP-ProtoSense are used primarily to obtain reproducible and spatially traceable evaluation labels rather than to define the inference rules of PartSense-IP. At test time, the framework does not require the input prototype to belong to one of the predefined deviation classes, and the final consistency score is computed from continuous part- and view-level measurements rather than from a closed-set deviation classifier. This design reduces, but does not eliminate, the dependence between the benchmark construction and the proposed scoring mechanism.

The principal failure modes observed on the real-captured challenge subset are associated with severe self-occlusion, incomplete or noisy depth measurements, highly reflective surfaces, very small decorative parts, and large appearance differences between stylized concept artwork and physical materials. Under these conditions, part correspondences may become uncertain and the predicted inconsistency response may extend beyond the human-annotated region. Compound real-world deviations can also produce gradual boundaries that are difficult to represent using a binary localization mask. These cases explain the remaining performance gap between the controlled synthetic and real-captured subsets and motivate larger-scale qualitative evaluation in future work.

## 5. Conclusions

This paper proposed PartSense-IP, a part-aware vision–language sensor fusion framework for visual–semantic consistency evaluation of IP prototypes. Different from conventional manual inspection or global image-level similarity comparison, the proposed framework formulates IP prototype evaluation as a sensor-captured consistency assessment task. Given a 2D concept image, an optional textual design description, and multi-view RGB-D observations of a prototype, PartSense-IP evaluates whether the prototype preserves the visual identity and semantic design intent of the original concept.

The proposed framework integrates multi-view RGB-D sensing, part-aware parsing, dense visual feature extraction, vision–language semantic alignment, and cross-view consistency modeling. A Part-Aware Visual–Semantic Consistency Fusion (PVCF) algorithm was developed to combine shape, color, part-level visual similarity, semantic consistency, and view stability into a unified IP consistency score. In addition to scalar scoring, the framework generates localized difference maps, 3D inconsistency visualization, and interpretable feedback reports, enabling designers to identify where the prototype deviates from the concept and which design attributes should be refined.

Experiments conducted under the IP-ProtoSense evaluation protocol demonstrate the effectiveness of the proposed method. PartSense-IP achieves stronger consistency scoring performance, higher inconsistency detection accuracy, and more accurate localization results than representative vision–language, dense-visual, segmentation-based, and 3D multimodal baselines. The ablation study confirms that part-aware parsing, semantic alignment, RGB-D sensing, cross-view stability, and PVCF fusion all contribute to the final performance. Robustness evaluations further show that the proposed method remains stable under reduced views, illumination changes, depth degradation, pose perturbation, occlusion, background clutter, and text-description noise. Moreover, the 3D-to-2D inconsistency map projection demonstrates that PartSense-IP can provide spatially interpretable feedback for practical prototype refinement.

## Figures and Tables

**Table 1:** Statistics of the proposed IP-ProtoSense evaluation dataset.

Item	Statistic
Source assets	300 assets, including 240 publicly available 3D assets and 60 self-designed concept–prototype pairs.
Concept references	300 concept images with textual design descriptions.
Prototype variants per concept	7 variants, comprising 1 clean prototype and 6 controlled inconsistent variants.
Controlled deviation types	Color shift, shape distortion, missing symbolic element, part displacement, texture inconsistency, and semantic identity loss.
Synthetic prototype samples	2100 samples.
Views per sample	6 views: front, left, right, back, top, and local close-up.
Synthetic RGB-D observations	12,600 RGB-D observations with corresponding camera-pose metadata.
Real-captured prototype samples	60 independently captured physical or 3D-printed prototype samples. These samples are excluded from training, validation, and parameter selection.
Annotation labels	Overall consistency score, deviation type, part-level label, and local inconsistency mask.
Number of annotators	3 annotators.
Score range	Five-point design-consistency ratings normalized to [0,1].
Data split	The synthetic assets are divided into 70% training, 10% validation, and 20% testing subsets. The 60 real-captured samples constitute an independent challenge subset.
Public 3D-asset sources	240 source assets collected from Objaverse, ABO, GSO, and OmniObject3D. Their canonical stylized front-view renderings are used as concept references.
Self-designed concept sources	60 source assets for which the original independently created 2D concept artwork is retained as the concept reference.

**Table 2:** Inter-rater agreement of the human annotations before consensus resolution.

Annotation Target	Agreement Metric	Result
Overall consistency rating	ICC (2, *k*)	0.886
Inconsistency category	Fleiss’ κ	0.817
Local inconsistency mask	Mean pairwise Dice	0.842

**Table 3:** Reproducibility settings of the pretrained models and baselines.

Method	Model Variant or Checkpoint	Feature or Output Used	Principal Settings
CLIP	OpenAI ViT-B/16	Final normalized image and text embeddings	224×224 input; cosine similarity; view-level average pooling.
DINOv2	ViT-B/14, pretrained LVD-142M checkpoint	Patch tokens from the final Transformer block	Class token excluded; masked average pooling; *ℓ*_2_ normalization.
SAM	ViT-H, sam_vit_h_4b8939.pth	Binary candidate part masks	Object-box-constrained automatic mask generation with a 32×32 point grid; predicted-IoU threshold 0.88; stability threshold 0.92; mask-NMS IoU threshold 0.70; minimum area 0.5%.
BLIP-2	Salesforce/blip2-opt-2.7b	Q-Former multimodal representation	Frozen visual encoder and Q-Former; view-level normalized similarity averaging.
Grounding DINO	Swin-T, groundingdino_swint_ogc.pth	Text-conditioned bounding boxes	Box threshold 0.35; text threshold 0.25; NMS IoU threshold 0.50.
ULIP	ULIP-PointBERT pretrained checkpoint	Point-cloud, image, and text embeddings	8192 sampled points; unit-sphere normalization; cosine similarity.
OpenShape	PointBERT-based OpenShape checkpoint	Open-world 3D multimodal embedding	8192 sampled colored points; unit-sphere normalization; global feature pooling.
PartSense-IP	Frozen SAM, DINOv2, and CLIP encoders	Shape, color, dense-visual, and semantic scores	α=(0.25,0.20,0.30,0.25); β=(0.80,0.20).
Part-CLIP	OpenAI ViT-B/16	Spatial Vision Transformer patch tokens	Same SAM masks; masked patch matching; uniform part and view averaging.
Part-DINOv2-MNN	DINOv2 ViT-B/14	Final-block patch tokens	Same SAM masks; mutual nearest-neighbor correspondence; uniform part and view averaging.
Part-ULIP	ULIP-PointBERT	Part-level point-cloud embeddings	Mask-to-point projection; 2048 points per visible part; uniform part and view averaging.

**Table 4:** Implementation details of the compared methods and the proposed PartSense-IP framework.

Item	Setting
Input RGB resolution	224×224 pixels for CLIP-, DINOv2-, and SAM-based feature extraction.
Depth processing	Valid-depth normalization, RGB-D back-projection, and pose-based multi-view fusion.
Concept input	A 2D concept image $I_{c}$ and an optional textual design description $T_{c}$.
View setting	Front, left, right, back, top, and local close-up views.
CLIP baseline	Global image–text and image–image cosine similarities averaged over all views.
DINOv2 baseline	Dense visual-feature similarities averaged over all views.
SAM + CLIP baseline	SAM-based part segmentation followed by CLIP-based part matching.
ULIP baseline	Point-cloud, image, and text alignment in a shared 3D multimodal feature space.
Part parsing	Promptable segmentation with small-mask filtering and part-level correspondence matching.
PVCF component weights	$\alpha_{1}=0.25$, $\alpha_{2}=0.20$, $\alpha_{3}=0.30$, and $\alpha_{4}=0.25$.
Part-identity weights	Fixed three-level concept-side rubric: $\omega_{k}=3$ for identity-defining parts, $\omega_{k}=2$ for major structural or appearance parts, and $\omega_{k}=1$ for secondary parts. Uniform weights are used when no part-specific identity cue is available.
View-stability weights	$\beta_{1}=0.80$ and $\beta_{2}=0.20$.
View importance	Larger weights are assigned to the front and local close-up views; the remaining views are used to evaluate cross-view stability.
Validation strategy	PVCF weights are selected using the validation split and remain fixed during testing.
Evaluation protocol	Deterministic methods are evaluated once using fixed inputs and parameters. Statistical uncertainty is estimated through 1000 asset-level bootstrap resamples. For stochastic robustness perturbations, results are averaged over three independently generated perturbation realizations.
Evaluation metrics	Pearson correlation, Spearman correlation, MAE, RMSE, accuracy, precision, recall, F1-score, mIoU, and pointing accuracy.

**Table 5:** Statistical comparison between PartSense-IP and the strongest competing baseline. Results are point estimates on the fixed test subset. The 95% confidence interval corresponds to the paired performance improvement estimated through 1000 asset-level bootstrap resamples.

Metric	Grounding DINO + CLIP	PartSense-IP	95% CI of Improvement	*p*-Value
Pearson correlation	0.928	0.965	[0.028,0.047]	<0.001
F1-score	0.899	0.940	[0.031,0.052]	<0.001
mIoU	0.687	0.760	[0.058,0.089]	<0.001
Pointing accuracy	0.847	0.910	[0.048,0.078]	<0.001

**Table 6:** Performance comparison on the controlled synthetic test subset and the independently captured real-world challenge subset. All model configurations, PVCF coefficients, decision thresholds, and preprocessing parameters are determined using only the synthetic training and validation subsets and are directly applied to the real-captured subset without further adjustment.

Evaluation Subset	Method	Pearson ↑	F1-Score ↑	mIoU ↑	Pointing Acc. ↑
Controlled synthetic test	CLIP-based matching	0.842	0.811	0.543	0.724
	DINOv2 matching	0.886	0.848	0.604	0.771
	SAM + CLIP	0.913	0.882	0.661	0.823
	ULIP	0.901	0.867	0.628	0.797
	PartSense-IP	0.965	0.940	0.760	0.910
Real-captured challenge	CLIP-based matching	0.748	0.716	0.421	0.617
	DINOv2 matching	0.781	0.752	0.466	0.658
	SAM + CLIP	0.806	0.779	0.508	0.701
	ULIP	0.793	0.764	0.487	0.680
	PartSense-IP	0.872	0.836	0.594	0.774

**Table 7:** Quantitative evaluation of the structured feedback generated by PartSense-IP. The predicted affected part and inconsistency type are compared with the existing human annotations.

Feedback-Quality Metric	Controlled Synthetic Test	Real-Captured Challenge
Affected-part accuracy ↑	0.918	0.842
Inconsistency-type accuracy ↑	0.904	0.817
Joint part–type accuracy ↑	0.861	0.758

**Table 8:** Backbone comparison under the same part-decomposition and multi-view aggregation protocol.

Method	Representation and Local Matching	Pearson ↑	F1-Score ↑	mIoU ↑	Pointing Acc. ↑
Part-CLIP	CLIP ViT patch-token matching	0.926	0.901	0.688	0.846
Part-DINOv2-MNN	DINOv2 mutual nearest-neighbor matching	0.940	0.915	0.713	0.869
Part-ULIP	Part-level RGB-D point-cloud matching	0.934	0.909	0.704	0.860
Full PartSense-IP	Multimodal PVCF with identity/view modeling	0.965	0.940	0.760	0.910

**Table 9:** Comparison of different fusion strategies using the same segmentation, DINOv2, CLIP, and RGB-D inputs.

Fusion Strategy	Part Correspondence	Identity-Aware Part Weighting	Cross-View Stability	Pearson ↑	F1-Score ↑	mIoU ↑	Pointing Acc. ↑
Global score averaging	No	No	No	0.892	0.873	0.646	0.806
Part-wise uniform fusion	Yes	No	No	0.931	0.908	0.702	0.856
Learned linear fusion	Yes	No	No	0.946	0.922	0.728	0.879
Full hierarchical PVCF	Yes	Yes	Yes	0.965	0.940	0.760	0.910

**Table 10:** Comparison of fixed, learned, and structure-aware fusion strategies. All methods use identical SAM-generated part masks, DINOv2 features, CLIP embeddings, RGB-D observations, part correspondences, and data splits.

Fusion Strategy	Learnable	Additional Parameters	Pearson ↑	F1-Score ↑	mIoU ↑	Pointing Acc. ↑	Explicit Component Diagnosis
Learned linear fusion	Yes	5	0.946	0.922	0.728	0.879	Yes
MLP fusion	Yes	705	0.957	0.934	0.746	0.898	No
Attention-based fusion	Yes	1.2 K	0.962	0.938	0.755	0.906	Partial
Transformer fusion	Yes	68.4 K	0.968	0.941	0.758	0.908	No
Graph-based part aggregation	Yes	24.7 K	0.966	0.943	0.763	0.912	Partial
Proposed fixed PVCF	No	0	0.965	0.940	0.760	0.910	Yes

**Table 11:** Sensitivity analysis of the PVCF component weights. The four coefficients correspond to shape consistency, color consistency, dense part-level visual similarity, and semantic consistency, respectively.

Fusion Setting	$\alpha_{1}$	$\alpha_{2}$	$\alpha_{3}$	$\alpha_{4}$	Pearson ↑	F1-Score ↑	mIoU ↑	Pointing Acc. ↑
Uniform fusion	0.25	0.25	0.25	0.25	0.958	0.933	0.748	0.901
Shape-emphasized	0.45	0.15	0.20	0.20	0.946	0.921	0.731	0.887
Color-emphasized	0.15	0.45	0.20	0.20	0.938	0.914	0.721	0.879
Visual-emphasized	0.20	0.15	0.45	0.20	0.960	0.935	0.752	0.904
Semantic-emphasized	0.20	0.15	0.20	0.45	0.951	0.928	0.739	0.893
Validation-learned fusion	0.24	0.18	0.32	0.26	0.967	0.942	0.764	0.912
Proposed fixed PVCF	0.25	0.20	0.30	0.25	0.965	0.940	0.760	0.910

**Table 12:** Robustness comparison under different sensing variations. The reported values are F1-scores for inconsistency detection. “Avg. F1” is averaged over the seven perturbed sensing conditions, excluding the clean setting, and “Drop” denotes the difference between the clean-setting F1-score and Avg. F1.

Method	F1-Score Under Sensing Perturbations								Robustness Summary	
	Clean Setting	Three Views	One View	Low Light	Strong Shadow	Depth Noise	Missing Depth	Pose Jitter	Avg. F1	Drop
CLIP-only	0.69	0.66	0.61	0.58	0.55	0.66	0.67	0.64	0.62	0.07
BLIP-2	0.76	0.72	0.65	0.67	0.62	0.70	0.71	0.68	0.68	0.08
DINOv2-only	0.79	0.75	0.68	0.69	0.64	0.73	0.74	0.70	0.70	0.09
SAM + CLIP	0.83	0.80	0.72	0.74	0.70	0.77	0.79	0.75	0.75	0.08
Grounding DINO + CLIP	0.88	0.85	0.78	0.80	0.77	0.82	0.83	0.80	0.81	0.07
ULIP	0.84	0.81	0.73	0.75	0.72	0.76	0.77	0.74	0.75	0.09
OpenShape	0.89	0.86	0.79	0.81	0.78	0.82	0.83	0.80	0.81	0.08
PartSense-IP	0.94	0.92	0.86	0.89	0.86	0.90	0.91	0.88	0.89	0.05

**Table 13:** Robustness comparison under practical perturbations. The reported values are F1-scores for inconsistency detection. All methods are evaluated using identical perturbation settings, input observations, and test samples. The best result in each row is highlighted in bold.

Perturbation Type	Condition	CLIP	BLIP-2	DINOv2	SAM + CLIP	GDINO + CLIP	ULIP	OpenShape	PartSense-IP
Occlusion/clutter	10% occlusion	0.63	0.67	0.74	0.80	0.85	0.78	0.82	**0.91**
Occlusion/clutter	20% occlusion	0.55	0.59	0.66	0.74	0.79	0.71	0.75	**0.88**
Occlusion/clutter	40% occlusion	0.42	0.46	0.54	0.62	0.68	0.58	0.63	**0.79**
Occlusion/clutter	Strong background clutter	0.50	0.54	0.61	0.69	0.75	0.65	0.70	**0.83**
Pose calibration error	5° rotation error	0.65	0.69	0.76	0.81	0.86	0.79	0.84	**0.91**
Pose calibration error	10° rotation error	0.60	0.63	0.70	0.76	0.81	0.73	0.78	**0.87**
Pose calibration error	20° rotation error	0.50	0.53	0.61	0.67	0.73	0.64	0.69	**0.80**
Pose calibration error	Pose translation jitter	0.58	0.61	0.68	0.73	0.78	0.71	0.75	**0.85**
Text-description noise	Incomplete description	0.64	0.68	0.75	0.79	0.84	0.78	0.80	**0.89**
Text-description noise	Noisy description	0.57	0.61	0.73	0.76	0.80	0.74	0.76	**0.84**
Text-description noise	Ambiguous description	0.54	0.58	0.70	0.73	0.76	0.72	0.73	**0.82**
Text-description noise	No text description	0.51	0.55	0.72	0.74	0.74	0.70	0.69	**0.81**
Average	All perturbations	0.56	0.60	0.68	0.74	0.78	0.71	0.75	**0.85**

## Data Availability

The IP-ProtoSense dataset metadata, annotations, training, validation, test splits, evaluation code, and generation and rendering scripts are available from the corresponding author upon reasonable request. Third-party source assets are subject to their original license conditions and may not be redistributed directly; corresponding asset identifiers and reconstruction information can be provided where applicable.
